# Effects of aquatic rehabilitation on symptoms, physical function, and quality of life in individuals with knee joint dysfunction: a meta-analysis

**DOI:** 10.3389/fphys.2026.1804926

**Published:** 2026-04-02

**Authors:** Yujie Jiang, Yueliang Peng

**Affiliations:** 1 School of Physical Education and Sports, Central China Normal University, Wuhan, China; 2 School of Education, Lincoln University College, Petaling Jaya, Malaysia

**Keywords:** aquatic rehabilitation, knee joint dysfunction, meta-analysis, osteoarthritis, physical function, quality of life, symptoms

## Abstract

**Background:**

Knee joint dysfunction, including osteoarthritis, ligament injury, and post-surgical conditions, impairs symptoms, physical function, and quality of life. Aquatic rehabilitation leverages water’s buoyancy, resistance, and hydrostatic properties to reduce joint load and facilitate exercise, but evidence on its effectiveness across populations and intervention parameters is inconsistent.

**Methods:**

We conducted a PRISMA-guided meta-analysis of randomized controlled trials (PROSPERO CRD420251139080) comparing structured aquatic exercise with land-based exercise or conventional treatment. Web of Science, PubMed, Embase, SPORTDiscus, CINAHL, and Cochrane Library were searched to July 2025. Change-score standardized mean differences (SMDs) with 95% confidence intervals (CIs) were pooled using random-effects models across symptoms, physical function, and quality of life. Heterogeneity was assessed using the I^2^ statistic. Pre-specified subgroup analyses examined disease type, age, session length, intervention duration, and training frequency. Risk of bias was assessed with RoB 2.0; evidence certainty was appraised using GRADE.

**Results:**

Twenty-nine trials (n = 1,984) were included. Aquatic rehabilitation significantly improved symptoms (SMD = −0.55, 95% CI: −0.73 to −0.38) and physical function (SMD = 0.50, 95% CI: 0.34 to 0.65) versus controls, while quality of life improvements were non-significant (SMD = 0.17, 95% CI: −0.15 to 0.50). Benefits were largest in patients with knee osteoarthritis and those <60 years. Interventions ≥8 weeks yielded greater symptom and functional gains. Functional subdomain analysis revealed pronounced improvements in balance, proprioception, and muscle strength, whereas mobility and flexibility showed smaller effects. Session length and training frequency had a minor influence. QoL improvements were primarily observed in younger participants.

**Conclusion:**

Aquatic rehabilitation effectively alleviates symptoms and enhances physical function in individuals with knee joint dysfunction, with the greatest benefits observed in knee osteoarthritis patients and adults younger than 60 years. Programs lasting at least 8 weeks yield optimal outcomes, particularly for balance, proprioception, and muscle strength. While improvements in quality of life are less consistent, younger participants may experience psychosocial gains. These findings support the integration of structured aquatic exercise into knee rehabilitation protocols, with attention to patient characteristics and program duration to maximize therapeutic effects.

**Systematic review registration:**

https://www.crd.york.ac.uk/PROSPERO/view/CRD420251139080, identifier CRD420251139080.

## 1 Introduction

Knee joint dysfunction is widely understood as a multifactorial condition that develops when structural or pathological changes in the joint interact with impairments in body functions, daily activities, and social participation (Fréz et al., 2024). The condition can arise from various causes, primarily osteoarthritis, ligament injury, and post-surgical complications, with patients experiencing persistent pain, stiffness, restricted range of motion, mechanical instability, and limitations in daily activities (Kolasinski et al., 2020). Global epidemiological data highlight the scale of this problem: knee osteoarthritis affects roughly 16% of adults, with an annual incidence of 203 cases per 10,000 people, showing higher prevalence in women (Cui et al., 2020). Since the mid-20th century, the prevalence has more than doubled, a trend linked to population aging and increasing obesity rates (Wallace et al., 2017).

Aquatic rehabilitation is a therapeutic exercise approach that uses the buoyancy, hydrostatic pressure, viscosity, and thermal properties of water to aid physical recovery (Becker, 2009). Compared to land-based training, this method allows patients to exercise with less joint stress and greater safety (Waller et al., 2014). The water environment provides multiple therapeutic benefits through its unique properties. The buoyancy reduces weight-bearing load during exercise, while hydrostatic pressure enhances stability and body awareness. Additionally, water resistance naturally facilitates strength and balance training (Bartels et al., 2016). These features make aquatic rehabilitation particularly suitable for individuals dealing with chronic pain, obesity, or postoperative restrictions, who often struggle with conventional rehabilitation programs (Wasser et al., 2017). Mechanistically, reduced mechanical loading lowers nociceptive input and pain, while the graded resistance and protected environment of the pool permit safe progressive strengthening and neuromuscular retraining—changes that directly underpin improvements in function and exercise tolerance (Tedeschi et al., 2024). Growing evidence has demonstrated the safety and effectiveness of aquatic rehabilitation in improving functional outcomes and exercise tolerance across various patient populations (Barker et al., 2014; Lei et al., 2024). By isolating the unique contribution of the aquatic environment beyond general exercise effects, this study clarifies when and why aquatic rehabilitation should be prioritized in knee care pathways.

Comprehensive rehabilitation outcome assessment traditionally focuses on three critical domains: symptoms, physical function, and health-related quality of life (QoL) (Küçükdeveci et al., 2011). For most patients, the primary concern is the alleviation of pain and stiffness, which commonly serves as the impetus for seeking medical care (King et al., 2025). As symptom resolution progresses, improvements in mobility and joint function emerge, contributing to enhanced independence and reduced risk of long-term disability (Rafiq et al., 2021). Because alleviating symptoms enables patients to participate more fully in exercise and reduces everyday limitations, clinical improvements in pain and function often extend beyond the physical domain to influence QoL (Ma et al., 2022). Aquatic rehabilitation, by combining pain relief with a safe environment for progressive training, thus addresses not only physical restoration but also the psychological and social dimensions of recovery (Tedeschi et al., 2024). QoL assessment extends beyond traditional clinical metrics, capturing the psychological and social dimensions of knee dysfunction that standard measures often overlook (Giotis et al., 2025). In practice, symptomatic relief frequently creates the conditions necessary for functional gains, and together these changes establish the foundation for enhanced daily well-being and higher overall QoL (Ma et al., 2022). This integrated approach to outcome assessment provides a holistic evaluation of rehabilitation success, encompassing both clinical efficacy and the practical impact on patients’ everyday lives.

Despite mounting evidence supporting the effectiveness of aquatic rehabilitation, current findings demonstrate considerable inconsistency in treatment outcomes (Assar et al., 2020; Lund et al., 2008; Munukka et al., 2020; Yesil et al., 2023). While several systematic reviews and meta-analyses have emerged, they reveal important methodological limitations and evidence gaps. Most syntheses have focused exclusively on osteoarthritis populations, overlooking patients with ligament injuries or post-surgical conditions who face similar functional challenges (Ma et al., 2022; Waller et al., 2014). Additionally, methodological limitations include insufficient consideration of baseline heterogeneity among included trials. Many existing meta-analyses synthesized post-intervention outcomes without adjusting for pre-intervention differences, a practice that may introduce systematic bias and yield potentially inflated treatment effects (Lu et al., 2015). The interpretation of therapeutic benefits is further confounded by heterogeneous control conditions across studies: some trials used land-based exercise as comparators (Batterham et al., 2011), others employed usual care or no-intervention controls (Waller et al., 2014), while certain studies combined different control types in their analyses (Song and Oh, 2022). Additionally, the substantial body of evidence published since 2020, including several large-scale randomized controlled trials, remains unintegrated into current systematic reviews, suggesting that existing clinical recommendations may not reflect contemporary evidence (Assar et al., 2020; Azizi et al., 2020; Hajouj et al., 2021; Jain and Shinde, 2024; Kalkhoran and Khanmohammadi, 2024; Khruakhorn and Chiwarakranon, 2021; Krishnan et al., 2021; Li et al., 2022; Munukka et al., 2020; Pipino et al., 2023; Rewald et al., 2020; Sandeep and Pooja, 2025; Slouma et al., 2025; Yesil et al., 2023). More importantly, prior meta-analyses have rarely examined the potential moderating factors that may explain variability in treatment effects. In particular, critical intervention parameters—such as exercise session length, total intervention duration, and training frequency—have seldom been systematically evaluated as potential effect modifiers. Likewise, population characteristics (e.g., disease type and age) and outcome subdomains (e.g., specific symptom types or functional components) may influence treatment responsiveness but have received limited attention in previous evidence syntheses. As a result, current evidence provides limited guidance regarding which patient populations benefit most from aquatic rehabilitation and under what intervention conditions optimal outcomes can be achieved.

To address these limitations and provide updated clinical guidance, we conducted a comprehensive meta-analysis of randomized controlled trials investigating the therapeutic effects of aquatic rehabilitation across the spectrum of knee joint dysfunction, including osteoarthritis, ligament injuries, and post-surgical conditions. Our analysis specifically focused on three key domains: symptom management, physical function restoration, and QoL. Importantly, beyond estimating overall pooled effects, we conducted a series of pre-specified subgroup analyses to explore potential moderators of treatment effectiveness. These analyses examined whether intervention effects varied according to disease type, participants’ mean age, and key intervention parameters, including exercise session length, total intervention duration, and training frequency. Additionally, we evaluated specific outcome subdomains within each primary outcome domain (e.g., symptom type and functional components) to better characterize differential treatment responses. By systematically investigating these potential effect modifiers, this study aims to provide more clinically informative evidence regarding when, for whom, and under what conditions aquatic rehabilitation may produce the greatest therapeutic benefits.

## 2 Methods

This study followed the Preferred Reporting Items for Systematic Reviews and Meta-Analyses (PRISMA) reporting guideline (Page et al., 2021). The protocol was prospectively registered in PROSPERO (CRD420251139080).

### 2.1 Search strategy

A systematic literature search was conducted in Web of Science, PubMed, Embase, SPORTDiscus, CINAHL, and the Cochrane Library from database inception to July 14, 2025. Only studies published in English were considered eligible. The final search was performed on July 14, 2025. To ensure comprehensive retrieval of eligible studies, we also examined the reference lists of relevant systematic reviews and meta-analyses identified during the initial search. The detailed search strategy is presented in **Supplementary Material 1**.

### 2.2 Study selection

The eligibility criteria were defined according to the PICO framework (Population, Intervention, Comparison, Outcomes).

Population (P): Individuals diagnosed with knee joint dysfunction, including degenerative or non-systemic knee disorders. Studies involving knee dysfunction secondary to systemic diseases (e.g., stroke, rheumatoid arthritis, hemophilic arthropathy) were excluded.

Intervention (I): Structured aquatic exercise programs conducted in water settings.

Comparison (C): Land-based exercise, usual care, health education, or no intervention. Trials in which aquatic interventions were used in the control group were excluded.

Outcomes (O): Knee-related symptoms, physical function, and quality of life assessed using validated instruments.

All retrieved records were first imported into EndNote 20, and duplicates were removed. Two independent reviewers (JYJ and PYL) screened titles and abstracts, followed by full-text evaluation against the predefined eligibility criteria. Disagreements were resolved through discussion and, when necessary, consultation with a third reviewer.

We included randomized controlled trials evaluating aquatic exercise interventions. Studies were excluded if they lacked knee-specific outcome data in multi-joint assessments, evaluated non-exercise aquatic therapies (e.g., passive immersion, Kneipp therapy), involved combined interventions without isolating aquatic exercise effects, or were non-full-text publications (conference abstracts, case reports, commentaries, editorials, or duplicate reports).

### 2.3 Data extraction

Data extraction was independently performed by two reviewers (JYJ and PYL) using a standardized form developed according to the Cochrane handbook (J. Higgins et al., 2019). The extracted information encompassed author names, publication year, participant characteristics (type of knee joint dysfunction, sample size, mean age, sex distribution), intervention details (exercise type, frequency, session length, total intervention period, water depth, water temperature), and comparison conditions. Outcome data were extracted for both intervention and control groups, including means and standard deviations at baseline and post-intervention timepoints, along with outcome measurement instruments.

When change scores were not directly reported, they were calculated using baseline and post-intervention means and standard deviations. If the standard deviation of change was not available, it was derived using the recommended formula from the Cochrane Handbook, assuming a pre–post correlation coefficient (r) of 0.5. Sensitivity analyses were conducted to examine the robustness of results to different plausible correlation values.

### 2.4 Risk of bias assessment

Risk of bias was assessed independently by two reviewers (JYJ and PYL) using the Cochrane RoB 2.0 tool across five domains (Sterne et al., 2019). Each was rated as low risk, some concerns, or high risk, with an overall judgment assigned. Disagreements were resolved through discussion, and if consensus was not reached, an independent researcher not involved in study selection or data extraction acted as an adjudicator. The results of the RoB 2.0 assessment informed the risk-of-bias domain in the GRADE evaluation of the certainty of evidence. The overall certainty of evidence for each outcome was appraised using the GRADE framework, considering risk of bias, inconsistency, indirectness, imprecision, and publication bias. The certainty of evidence was classified as high, moderate, low, or very low.

### 2.5 Data analysis

Effects were analyzed using change scores from baseline to post-intervention. Meta-analyses were performed using Review Manager (version 5.3; Cochrane Collaboration) and Stata (version 17.0; StataCorp). Effect sizes were estimated with standardized mean differences (SMDs) and 95% confidence intervals (CIs). All effect sizes were directionally standardized so that negative values consistently indicated improvement in favor of aquatic rehabilitation. The analyses were structured around three primary outcome domains: (1) symptoms, including pain and stiffness; (2) physical function, encompassing balance, mobility, flexibility, proprioception, and muscle strength; and (3) QoL. Pre-specified subgroup analyses were conducted to explore potential sources of heterogeneity according to control group type (land-based exercise vs. conventional treatment), disease type, participants’ mean age, intervention characteristics (session length, total intervention duration, and training frequency), as well as specific outcome subdomains within each primary domain (e.g., symptom type and functional component).

Given the anticipated clinical and methodological heterogeneity across studies (including variations in intervention protocols, participant characteristics, and outcome measurements), we applied a random-effects model for all meta-analyses. Between-study heterogeneity was quantified using the *I^2^* statistic, with values of 25%, 50%, and 75% suggesting low, moderate, and high heterogeneity, respectively (Higgins et al., 2003).

The robustness of our findings was assessed through comprehensive sensitivity analyses. Using Stata, we performed leave-one-out analyses where each study was sequentially excluded to evaluate its influence on the pooled estimates and heterogeneity. Additionally, studies with effect sizes beyond three standard deviations from the pooled estimate were identified as statistical outliers. Influence analyses were conducted by comparing pooled estimates with and without these identified studies to assess their impact on the overall results. Publication bias was evaluated through visual inspection of funnel plots and quantified using Egger’s regression test, with *p* < 0.05 indicating potential publication bias.

## 3 Results

### 3.1 Selection of studies

**Figure 1:**
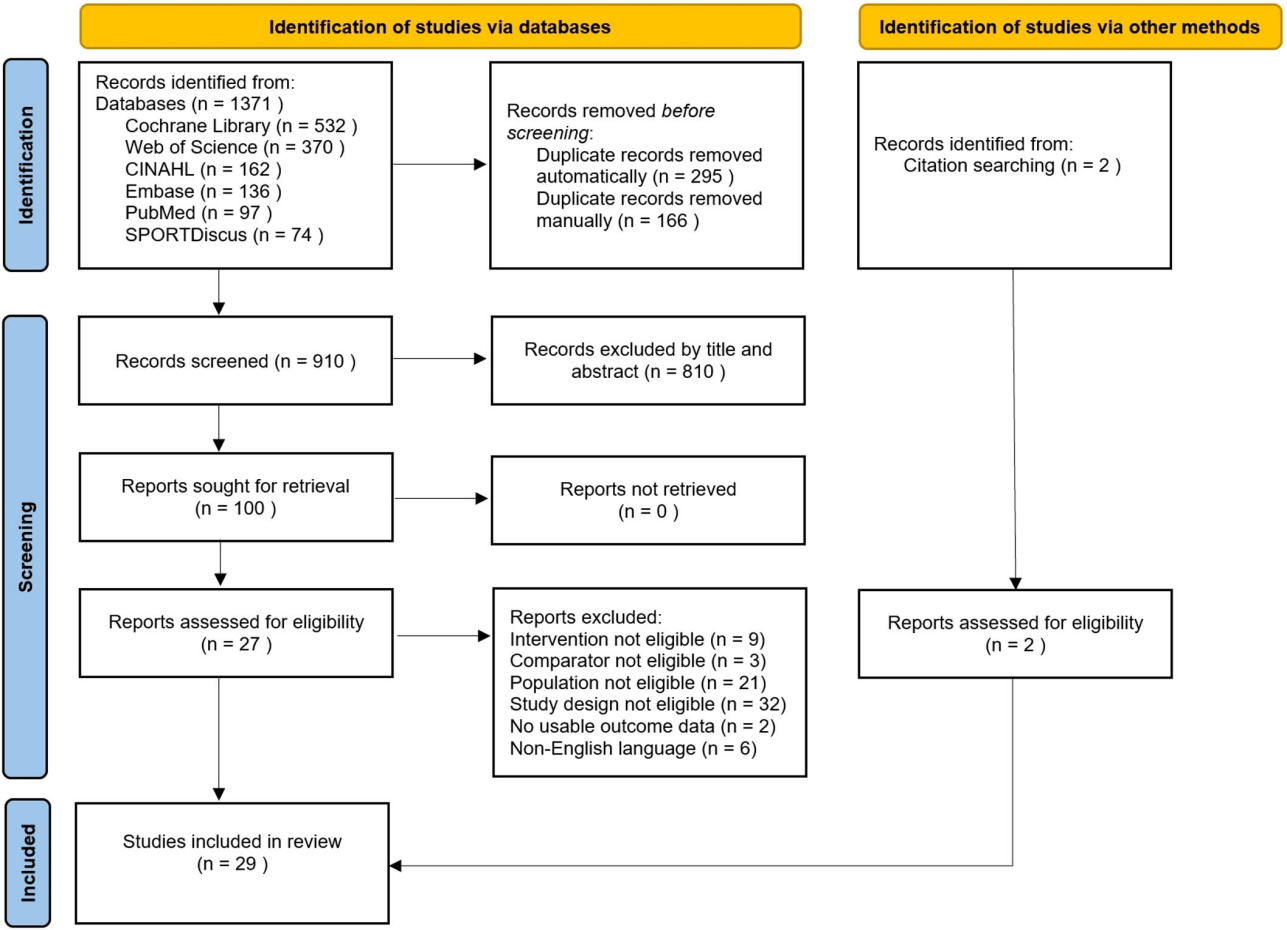
Flow diagram of the process of literature identification.

The selection process is presented in **Figure 1**. A total of 1,371 records were initially identified, of which 910 remained after removing duplicates. Following title and abstract screening, 100 full-text articles were assessed for eligibility. Ultimately, 27 studies from database searches and 2 from citation tracking were included in the systematic review.

### 3.2 Study characteristics

**Supplementary Material 2**, **Supplementary Table 1** summarizes the characteristics of the 29 studies included in the meta-analysis. The meta-analysis encompassed 1,984 participants from included trials, with individual study sample sizes ranging from 13 to 290 participants. The study populations represented diverse clinical presentations of knee dysfunction, including knee osteoarthritis (KOA) (Assar et al., 2020; Azizi et al., 2020; Dias et al., 2017; Jain and Shinde, 2024; Kalkhoran and Khanmohammadi, 2024; Khruakhorn and Chiwarakranon, 2021; Krishnan et al., 2021; Kuptniratsaikul et al., 2019; Lim et al., 2010; Lund et al., 2008; Munukka et al., 2016, 2020; Rewald et al., 2020; Sandeep and Pooja, 2025; Silva et al., 2008; Slouma et al., 2025; Taglietti et al., 2018; Tamin and Loekito, 2018; Waller et al., 2014; Wang et al., 2011; Wyatt et al., 2001; Yennan et al., 2010), anterior cruciate ligament reconstruction (ACLR) (Hajouj et al., 2021; Li et al., 2022; Pipino et al., 2023; Zamarioli et al., 2008), arthroscopic partial meniscectomy (APM) (Yesil et al., 2023), total knee replacement (TKR) (Harmer et al., 2009), and unicompartmental knee replacement (UKR) (Valtonen et al., 2010). The average age of participants ranged from 24.2 years to 70.8 years.

The aquatic rehabilitation programs varied considerably in content, intensity, and duration. Session length ranged from 30 to 90 minutes, delivered two to four times per week, over periods of 3 to 18 weeks. Most interventions combined multiple modalities such as stretching, resistance, aerobic, balance, proprioceptive, and step exercises, and some adopted progressive or high-intensity resistance protocols. Water depth was generally waist to chest level, with temperatures between 25 °C and 36 °C. Comparator groups also differed, including land-based training, conventional care, education programs, or no intervention.

Across the included trials, outcomes clustered into three main domains: symptoms, physical function, and QoL. Symptoms were evaluated in 27 studies, through pain scales—Western Ontario and McMaster Universities Osteoarthritis Index (WOMAC) pain subscale, Knee Injury and Osteoarthritis Outcome Score (KOOS) pain subscale, 36-Item Short Form Survey (SF-36) pain subscale, Visual Analogue Scale, Numeric Rating Scale, or Brief Pain Inventory. Physical function was reported in 29 studies, assessed through daily activity questionnaires such as the WOMAC function subscale, KOOS activities of daily living subscale, SF-36 physical functioning subscale, and Lower Extremity Functional Scale; sport-specific instruments including the KOOS sport/recreation subscale, International Knee Documentation Committee subjective knee form, and Lysholm Knee Scoring Scale; and objective performance tests such as balance (Berg Balance Scale, Timed Up and Go test), mobility (6-Minute Walk Test, stair climb, timed walk), flexibility (Range of Motion), proprioception (joint position sense), and muscle strength (isokinetic dynamometry, one-repetition maximum leg press, 5-times sit-to-stand test, and chair stand test). QoL was reported in seven studies, most commonly measured with the KOOS QoL subscale.

Some secondary or less frequently assessed outcomes (e.g., swelling or general health scores) were not synthesized quantitatively because fewer than three studies provided sufficient data.

### 3.3 Meta-analysis

#### 3.3.1 Overall pooled effects

Overall pooled analyses were conducted to evaluate the effects of aquatic rehabilitation on three primary outcome domains: symptoms, physical function, and QoL. For symptoms, the pooled results demonstrated a statistically significant improvement in favor of aquatic rehabilitation compared with control conditions (SMD = −0.55, 95% CI: −0.73 to −0.38, *p* < 0.001). However, substantial between-study heterogeneity was observed (*I^2^* = 87%). For physical function, aquatic rehabilitation was also associated with a significant positive effect (SMD = 0.50, 95% CI: 0.34 to 0.65, *p* < 0.001), although heterogeneity remained high across the included studies (*I^2^* = 90%). In contrast, the pooled effect for quality of life (QoL) was not statistically significant (SMD = 0.17, 95% CI: −0.15 to 0.50, *p* = 0.30), with moderate to substantial heterogeneity detected among studies (*I^2^* = 74%). Forest plots illustrating the pooled effects for these outcomes are presented in **Supplementary Appendix 3** (**Supplementary Figures S1**-**S3**).

#### 3.3.2 Subgroup analyses

To explore potential sources of heterogeneity and identify clinically relevant moderators of treatment effects, pre-specified subgroup analyses were conducted according to control type, disease type, participants’ mean age, intervention characteristics (session length, total intervention duration, and training frequency), and outcome subdomains within each primary outcome domain. The results of subgroup analyses are presented in **Tables 1**–**3**; **Supplementary Material 4** (**Supplementary Figures S4**–**S23**).

**Table 1:** Subgroup analyses of aquatic rehabilitation effects on symptoms.

Subgroup	Categories	*k*	SMD (95%CI)	Test for overall effect: *p*	Test for subgroup differences: *p*
Type of control group	CTG	28	-0.73 [-0.96, -0.50]	<0.001	0.08
	LBG	39	-0.43 [-0.68, -0.17]	<0.001	
Population	KOA	57	-0.63 [-0.83, -0.43]	<0.001	<0.001
	ACLR	3	-0.07 [-0.53, 0.40]	0.77	
	TKR	3	0.06 [-0.16, 0.29]	0.58	
	UKR	2	-0.33 [-0.73, 0.06]	0.10	
	APM	2	-0.31 [-0.82, 0.20]	0.23	
Age, year	<60	27	-0.84 [-1.18, -0.49]	<0.001	<0.05
	≥60	39	-0.40 [-0.59, -0.21]	<0.001	
Session length, min	<60	30	-0.52 [-0.81, -0.22]	<0.001	0.51
	≥60	34	-0.64 [-0.86, -0.42]	<0.001	
Duration, weeks	<8	15	-0.17 [-0.35, 0.00]	0.05	<0.001
	≥8	52	-0.67 [-0.89, -0.45]	<0.001	
Frequency, times/week	<3	25	-0.47 [-0.74, -0.21]	<0.001	0.48
	≥3	42	-0.60 [-0.84, -0.36]	<0.001	
Type of symptoms	Pain	50	-0.61 [-0.84, -0.39]	<0.001	0.57
	Stiffness	8	-0.46 [-0.92, -0.01]	0.05	

CTG, conventional treatment group; LBG, land-based training group; KOA, knee osteoarthritis; ACLR, anterior cruciate ligament reconstruction; TKR, total knee replacement; UKR, unilateral knee replacement; APM, arthroscopic partial meniscectomy.

**Table 2:** Subgroup analyses of aquatic rehabilitation effects on physical function.

Subgroup	Categories	*k*	SMD (95%CI)	Test for overall effect: *p*	Test for subgroup differences: *p*
Type of control group	CTG	43	0.46 [0.31, 0.61]	<0.001	0.71
	LBG	64	0.51 [0.27, 0.76]	<0.001	
Population	KOA	74	0.58 [0.37, 0.78]	<0.001	<0.001
	ACLR	11	0.40 [-0.14, 0.94]	0.14	
	TKR	5	-0.03 [-0.21, 0.14]	0.72	
	UKR	5	0.52 [0.26, 0.77]	<0.001	
	APM	4	-0.07 [-0.43, 0.28]	0.69	
Age, year	<60	49	0.85 [0.55, 1.15]	<0.001	<0.001
	≥60	56	0.24 [0.12, 0.36]	<0.001	
Session length, min	<60	52	0.70 [0.44, 0.96]	<0.001	<0.05
	≥60	49	0.35 [0.19, 0.52]	<0.001	
Duration, weeks	<8	36	0.25 [0.10, 0.41]	<0.01	<0.01
	≥8	71	0.63 [0.41, 0.84]	<0.001	
Frequency, times/week	<3	39	0.36 [0.17, 0.55]	<0.001	0.16
	≥3	68	0.57 [0.36, 0.79]	<0.001	
Type of physical function	Balance	7	0.68 [0.15, 1.21]	<0.05	<0.01
	Mobility	17	0.25 [-0.01, 0.50]	0.06	
	Flexibility	16	0.77 [-0.00, 1.53]	0.05	
	Proprioception	6	1.18 [0.73, 1.62]	<0.001	
	Muscle strength	24	0.33 [0.15, 0.50]	<0.001	

CTG, conventional treatment group; LBG, land-based training group; KOA, knee osteoarthritis; ACLR, anterior cruciate ligament reconstruction; TKR, total knee replacement; UKR, unilateral knee replacement; APM, arthroscopic partial meniscectomy.

**Table 3:** Subgroup analyses of aquatic rehabilitation effects on quality of life.

Subgroup	Categories	*k*	SMD (95%CI)	Test for overall effect: *p*	Test for subgroup differences: *p*
Type of control group	CTG	6	0.34 [0.01, 0.66]	0.77	0.24
	LBG	4	-0.09 [-0.73, 0.54]	0.05	
Population	KOA	10	0.17 [-0.15, 0.50]	0.30	
Age, year	<60	3	0.78 [0.43, 1.13]	<0.001	<0.001
	≥60	7	-0.08 [-0.38, 0.21]	0.58	
Session length, min	<60	3	-0.09 [-0.85, 0.67]	0.81	0.38
	≥60	7	0.29 [-0.09, 0.67]	0.14	
Duration, weeks	<8	1	-0.50 [-1.07, 0.06]	0.08	<0.05
	≥8	9	0.25 [-0.09, 0.58]	0.15	
Frequency, times/week	<3	5	0.33 [-0.32, 0.99]	0.31	0.46
	≥3	5	0.06 [-0.25, 0.37]	0.71	

CTG, conventional treatment group; LBG, land-based training group; KOA, knee osteoarthritis.

##### 3.3.2.1 Symptoms

When stratified by control type, aquatic rehabilitation demonstrated significant improvements compared with both conventional treatment and land-based training groups. Specifically, the effect size for studies comparing aquatic rehabilitation with conventional treatment was moderate and statistically significant (SMD = −0.73, 95% CI: −0.96 to −0.50, *p* < 0.001), while comparisons with land-based training yielded a still significant effect (SMD = −0.43, 95% CI: −0.68 to −0.17, *p* < 0.001), with no statistically significant difference between the subgroups (*p* = 0.08). When examining specific symptom subdomains, aquatic rehabilitation significantly reduced pain (SMD = −0.61, 95% CI: −0.84 to −0.39, p < 0.001) and stiffness (SMD = −0.46, 95% CI: −0.92 to −0.01, p = 0.05), with no statistically significant difference between these subdomains (p = 0.57).

Analysis by disease type revealed that the largest and most robust effects were observed in patients with KOA (SMD = −0.63, 95% CI: −0.83 to −0.43, *p* < 0.001). In contrast, aquatic rehabilitation had non-significant effects in patients who underwent ACLR (SMD = −0.07, 95% CI: −0.53 to 0.40, *p* = 0.77), TKR (SMD = 0.06, 95% CI: −0.16 to 0.29, p = 0.58), UKR (SMD = −0.33, 95% CI: −0.73 to 0.06, *p* = 0.10), or APM (SMD = −0.31, 95% CI: −0.82 to 0.20, *p* = 0.23), with a statistically significant difference among disease types (*p* < 0.001), indicating that the overall benefit is largely driven by KOA populations. Regarding participants’ mean age, individuals under 60 years experienced greater symptom improvement (SMD = −0.84, 95% CI: −1.18 to −0.49, *p* < 0.001) compared with participants more than 60 years old (SMD = −0.40, 95% CI: −0.59 to −0.21, *p* < 0.001), with a significant difference between age subgroups (p < 0.05).

Subgroup analyses based on session length showed improvements for both shorter (<60 min; SMD = −0.52, 95% CI: −0.81 to −0.22, *p* < 0.001) and longer sessions (≥60 min; SMD = −0.64, 95% CI: −0.86 to −0.42, *p* < 0.001), with no significant difference observed between these groups (*p* = 0.51). In contrast, total intervention duration demonstrated a pronounced effect, with interventions lasting ≥8 weeks yielding substantially larger improvements (SMD = −0.67, 95% CI: −0.89 to −0.45, *p* < 0.001) compared with shorter programs (<8 weeks; SMD = −0.17, 95% CI: −0.35 to 0.00, *p* = 0.05), and the difference between these subgroups was statistically significant (*p* < 0.001). Training frequency showed comparable improvements for lower (<3 sessions/week; SMD = −0.47, 95% CI: −0.74 to −0.21, *p* < 0.001) and higher frequencies (≥3 sessions/week; SMD = −0.60, 95% CI: −0.84 to −0.36, *p* < 0.001), with no significant difference between groups (*p* = 0.48).

##### 3.3.2.2 Physical function

When stratified by control type, aquatic rehabilitation significantly improved physical function compared with both conventional treatment (SMD = 0.46, 95% CI: 0.31 to 0.61, *p* < 0.001) and land-based training groups (SMD = 0.51, 95% CI: 0.27 to 0.76, *p* < 0.001), with no significant difference between the subgroups (*p* = 0.71). Examination of specific physical function subdomains revealed significant improvements in balance (SMD = 0.68, 95% CI: 0.15 to 1.21, *p* < 0.05), proprioception (SMD = 1.18, 95% CI: 0.73 to 1.62, *p* < 0.001), and muscle strength (SMD = 0.33, 95% CI: 0.15 to 0.50, *p* < 0.001), while effects for mobility (SMD = 0.25, 95% CI: −0.01 to 0.50, *p* = 0.06) and flexibility (SMD = 0.77, 95% CI: −0.00 to 1.53, *p* = 0.05) were not statistically significant. The overall difference among physical function subdomains was statistically significant (*p* < 0.01), indicating that the type of functional outcome may contribute to heterogeneity.

Analysis by disease type demonstrated the most robust improvements in patients with KOA (SMD = 0.58, 95% CI: 0.37 to 0.78, *p* < 0.001) and UKR (SMD = 0.52, 95% CI: 0.26 to 0.77, *p* < 0.001), whereas effects were non-significant in patients with ACLR (SMD = 0.40, 95% CI: −0.14 to 0.94, *p* = 0.14), TKR (SMD = −0.03, 95% CI: −0.21 to 0.14, *p* = 0.72), and APM (SMD = −0.07, 95% CI: −0.43 to 0.28, *p* = 0.69), with a statistically significant difference among disease types (*p* < 0.001), suggesting that diagnosis is a major contributor to heterogeneity. Regarding participants’ mean age, individuals under 60 years demonstrated greater improvements in physical function (SMD = 0.85, 95% CI: 0.55 to 1.15, *p* < 0.001) than those 60 years or older (SMD = 0.24, 95% CI: 0.12 to 0.36, *p* < 0.001), with a significant difference between age subgroups (*p* < 0.001).

Subgroup analyses based on session length showed significant improvements for both shorter sessions (<60 min; SMD = 0.70, 95% CI: 0.44 to 0.96, *p* < 0.001) and longer sessions (≥60 min; SMD = 0.35, 95% CI: 0.19 to 0.52, *p* < 0.001). A significant difference between subgroups was observed (*p* < 0.05), indicating that interventions with shorter session durations (<60 min) were associated with larger improvements in physical function. Similarly, total intervention duration had a pronounced impact, with interventions lasting ≥8 weeks yielding greater improvements (SMD = 0.63, 95% CI: 0.41 to 0.84, *p* < 0.001) than those <8 weeks (SMD = 0.25, 95% CI: 0.10 to 0.41, *p* < 0.01), and the difference was statistically significant (*p* < 0.01). Training frequency showed significant effects for both lower (<3 sessions/week; SMD = 0.36, 95% CI: 0.17 to 0.55, *p* < 0.001) and higher frequencies (≥3 sessions/week; SMD = 0.57, 95% CI: 0.36 to 0.79, *p* < 0.001), with no significant difference between subgroups (*p* = 0.16).

##### 3.3.2.3 QoL

When stratified by control type, aquatic rehabilitation demonstrated a marginally significant improvement in quality of life compared with conventional treatment (SMD = 0.34, 95% CI: 0.01 to 0.66, *p* = 0.05). In contrast, the comparison with land-based training showed a non-significant effect (SMD = −0.09, 95% CI: −0.73 to 0.54, *p* = 0.77). No statistically significant difference between the two control types was observed (*p* = 0.24).

All included studies assessing QoL involved patients with KOA, yielding an overall non-significant effect (SMD = 0.17, 95% CI: −0.15 to 0.50, *p* = 0.30). Analysis by participants’ mean age revealed significant improvements in younger patients (<60 years; SMD = 0.78, 95% CI: 0.43 to 1.13, *p* < 0.001), whereas effects were non-significant in those aged ≥60 years (SMD = −0.08, 95% CI: −0.38 to 0.21, *p* = 0.58), with a statistically significant difference between these age subgroups (*p* < 0.001).

Subgroup analyses based on session length showed non-significant effects for both shorter (<60 min; SMD = −0.09, 95% CI: −0.85 to 0.67, *p* = 0.81) and longer sessions (≥60 min; SMD = 0.29, 95% CI: −0.09 to 0.67, p = 0.14), with no significant difference between subgroups (*p* = 0.38). Regarding total intervention duration, neither subgroup showed a statistically significant effect. Interventions lasting ≥8 weeks yielded an effect size of SMD = 0.25 (95% CI: −0.09 to 0.58, p = 0.15), whereas programs shorter than 8 weeks showed an effect size of SMD = −0.50 (95% CI: −1.07 to 0.06, p = 0.08). However, the difference between the two subgroups was statistically significant (p < 0.05). Training frequency showed non-significant effects for both lower (<3 sessions/week; SMD = 0.33, 95% CI: −0.32 to 0.99, *p* = 0.31) and higher frequencies (≥3 sessions/week; SMD = 0.33, 95% CI: −0.32 to 0.99, *p* = 0.71), with no significant difference between subgroups (*p* = 0.46).

### 3.4 Sensitivity analysis

Leave-one-out analyses in Stata showed that omitting individual studies did not alter the direction or significance of the pooled effects, supporting the robustness of the findings. Forest plot inspection and influence diagnostics in RevMan identified four outliers. For composite symptoms, excluding Sandeep and Pooja, 2025 (1) and Taglietti et al., 2018 (1) slightly reduced the pooled effect, but it remained significant (SMD = −0.40, 95% CI: −0.51 to −0.29, *p* < 0.001), with *I^2^* decreasing from 87% to 69%. For composite physical function, excluding Sandeep and Pooja, 2025 (1–3) yielded a modestly smaller but still significant effect (SMD = 0.34, 95% CI: 0.24 to 0.44, *p* < 0.001), and *I^2^* declined from 90% to 75%. Overall, the results were stable, although certain studies contributed notably to heterogeneity.

### 3.5 Quality assessment and publication bias

The methodological quality assessment of included studies is presented in **Supplementary Figure S24**, **S25**, **Supplementary Table S2** (GRADE evidence profiles) in **Supplementary Material 5**. The funnel plots showed symmetric distribution for all three outcome domains, suggesting no obvious publication bias. Egger’s test results were consistent with the visual assessment for physical function (*p* = 0.589) and QoL (*p* = 0.706). For the symptoms domain, while the initial Egger’s test indicated potential publication bias (*p* = 0.021), sensitivity analysis after removing two identified outliers demonstrated no significant bias (*p* = 0.622). Detailed results are provided in the **Supplementary Material 5**, **Supplementary Figures S26**–**S28**.

## 4 Discussion

The present systematic review and meta-analysis synthesized evidence from randomized controlled trials to evaluate the effects of aquatic rehabilitation on symptoms, physical function, and quality of life in individuals with knee joint dysfunction. Overall, the findings indicate that aquatic rehabilitation provides beneficial effects across these three clinically important domains, supporting its role as an effective rehabilitation strategy for individuals with knee-related impairments. Importantly, beyond estimating overall treatment effects, this study systematically explored several potential sources of heterogeneity through pre-specified subgroup analyses. By examining population characteristics (e.g., disease type and age), intervention parameters (e.g., session length, total intervention duration, and training frequency), and specific outcome subdomains within each primary outcome domain, the present analysis provides a more nuanced understanding of how aquatic rehabilitation effects may vary across different clinical and intervention contexts. These findings contribute to the growing body of evidence by moving beyond the question of whether aquatic rehabilitation is effective to addressing the clinically relevant question of for whom and under what intervention conditions aquatic rehabilitation may yield the greatest therapeutic benefit.

Our overall pooled analyses demonstrated that aquatic rehabilitation significantly improved both symptoms (SMD = −0.55, 95% CI: −0.73 to −0.38) and physical function (SMD = 0.50, 95% CI: 0.34 to 0.65) compared with control conditions, whereas the effect on quality of life was smaller and non-significant (SMD = 0.17, 95% CI: −0.15 to 0.50). These results indicate that the primary benefit of aquatic rehabilitation lies in alleviating pain and stiffness and enhancing functional capacity, which are clinically meaningful outcomes directly relevant to daily activities and mobility. The modest impact on quality of life may reflect the multifactorial nature of QoL, which is influenced not only by physical recovery but also by psychological, social, and environmental factors (Farr Ii et al., 2013). Nonetheless, the consistent positive effects on core clinical domains underscore aquatic rehabilitation as a safe and effective intervention, offering measurable improvements over both conventional treatment and land-based exercise. Moreover, these findings highlight the importance of interpreting QoL outcomes in context, suggesting that functional gains and symptom relief may precede observable improvements in broader well-being measures, particularly in heterogeneous patient populations.

The subgroup analyses for symptoms revealed several clinically meaningful moderators of treatment effects. First, the comparison with different control types showed that aquatic rehabilitation significantly reduced symptoms when compared with both conventional treatment and land-based exercise, and no statistically significant difference was observed between these two comparator categories. This suggests that the therapeutic effects of aquatic rehabilitation extend beyond the absence of treatment and remain comparable to structured land-based exercise programs. Previous meta-analyses have reported similar findings, indicating that aquatic exercise is at least as effective as land-based rehabilitation in improving pain outcomes in individuals with knee disorders (Barker et al., 2014; Waller et al., 2014). One possible explanation is that both aquatic and land-based programs incorporate therapeutic movement and muscle activation, which contribute to symptom improvement. However, the aquatic environment may provide additional benefits through buoyancy-assisted unloading and hydrostatic pressure, which reduce joint compression and nociceptive input during movement, thereby facilitating pain relief even in patients with limited tolerance for weight-bearing activity (Becker, 2009). Disease type emerged as a significant moderator of symptom outcomes. The most pronounced benefits were observed in patients with KOA, whereas the effects were smaller and non-significant in populations undergoing ACL reconstruction, knee replacement, or meniscectomy. This pattern likely reflects fundamental differences in disease pathology and rehabilitation needs. KOA is a chronic degenerative condition characterized by persistent pain, stiffness, and joint loading sensitivity, making it particularly responsive to interventions that reduce mechanical stress during exercise (Hunter et al., 2020). Aquatic exercise has been shown to alleviate pain and disability in patients with knee or hip osteoarthritis, likely because the buoyant aquatic environment reduces joint loading while allowing patients to maintain active movement (Bartels et al., 2016). In contrast, post-surgical populations often experience symptoms related to tissue healing, surgical trauma, and neuromuscular deficits rather than chronic joint degeneration. As a result, symptom improvement in these populations may depend more heavily on targeted neuromuscular retraining or strength restoration than on load-reducing exercise modalities alone (Adams et al., 2012). Participant age also significantly moderated symptom outcomes, with individuals younger than 60 years demonstrating greater improvements. Several mechanisms may explain this finding. Younger patients typically exhibit higher levels of physical activity, greater muscle plasticity, and faster neuromuscular adaptation, which may enhance responsiveness to exercise-based interventions (Franchi et al., 2017). In addition, younger individuals may experience fewer comorbidities and better baseline functional capacity, enabling them to participate more actively in rehabilitation programs. Similar age-related differences in treatment responsiveness have been reported in previous rehabilitation studies (Ma et al., 2022). By contrast, older populations may experience more advanced structural degeneration, chronic pain sensitization, and reduced physiological reserve, all of which may limit the magnitude of symptom improvement achievable through exercise interventions alone (Hunter et al., 2020). Among intervention characteristics, total intervention duration appeared to be the most influential factor. Programs lasting at least eight weeks produced substantially larger symptom reductions than shorter interventions, suggesting that sustained exposure to aquatic exercise is necessary to achieve meaningful clinical benefits. This observation aligns with previous studies indicating that exercise-induced neuromuscular adaptations and pain modulation mechanisms typically require several weeks of consistent training to develop (Gabriel et al., 2006). In contrast, session length and training frequency did not significantly moderate symptom outcomes. One possible explanation is that pain reduction may depend more strongly on cumulative exercise exposure and progressive adaptation than on the specific structure of individual sessions. As long as the intervention is delivered regularly and maintained over sufficient duration, variations in session length or weekly frequency may exert relatively minor influence on overall symptom relief.

Subgroup analyses of physical function also revealed several factors that may contribute to heterogeneity in treatment effects. Similar to symptom outcomes, aquatic rehabilitation significantly improved physical function compared with both conventional treatment and land-based exercise, and no difference between these control types was observed. This finding is broadly consistent with previous meta-analyses reporting comparable functional improvements between aquatic and land-based exercise programs (Lu et al., 2015; Song and Oh, 2022). However, the present study extends previous evidence by examining specific functional subdomains rather than relying solely on composite functional indices. When functional outcomes were disaggregated, aquatic rehabilitation demonstrated significant improvements in balance, proprioception, and muscle strength, whereas effects on mobility and flexibility were not statistically significant. These results may reflect the distinctive biomechanical properties of the aquatic environment. Water viscosity and turbulence continuously challenge postural control, requiring ongoing neuromuscular adjustments that stimulate proprioceptive and balance adaptations (Becker, 2009). At the same time, water resistance provides moderate but consistent muscular loading that can enhance muscular endurance and moderate strength development (Hall et al., 1998). However, improvements in mobility and flexibility may depend more strongly on joint-specific range-of-motion exercises or high-intensity strengthening protocols typically performed in land-based settings (Fransen et al., 2015). Disease type again emerged as a significant moderator of functional outcomes, with the most robust improvements observed in patients with KOA and UKR. These findings suggest that aquatic rehabilitation may be particularly beneficial for individuals experiencing chronic functional limitations due to degenerative joint changes (Hunter et al., 2020). In contrast, functional recovery in ACL reconstruction or meniscectomy patients often requires sport-specific neuromuscular training, plyometric exercises, and progressive loading strategies that may not be fully replicated in aquatic environments (Adams et al., 2012). Age also played a significant moderating role in functional outcomes. Participants younger than 60 years exhibited substantially larger improvements than older adults. Younger individuals generally possess greater neuromuscular plasticity, higher baseline physical capacity, and greater tolerance for progressive exercise intensity (Franchi et al., 2017). In contrast, older adults may experience sarcopenia, balance impairments, and comorbid conditions that limit the magnitude of functional gains achievable through rehabilitation programs (Cruz-Jentoft et al., 2019). These findings highlight the importance of tailoring aquatic rehabilitation protocols to patient characteristics and combining them with complementary training modalities when necessary. Interestingly, session length emerged as a significant moderator for functional outcomes, with shorter sessions (<60 min) producing larger improvements than longer sessions. This finding may appear counterintuitive but could reflect greater adherence and reduced fatigue during shorter sessions, particularly among patients with pain or limited endurance. In addition, shorter sessions may allow therapists to maintain higher exercise intensity or more focused task-specific training, thereby promoting more effective neuromuscular adaptations (Gabriel et al., 2006). Total intervention duration also significantly influenced functional outcomes, with programs lasting eight weeks or longer producing greater improvements. These results reinforce the importance of sustained training exposure for achieving meaningful functional gains.

The subgroup analyses for quality of life revealed a more complex pattern of findings. Overall, aquatic rehabilitation did not produce a statistically significant improvement in QoL, although a marginally significant effect was observed when compared with conventional treatment. This finding partially aligns with previous meta-analyses reporting inconsistent or modest effects of aquatic exercise on QoL outcomes (Barker et al., 2014; Bartels et al., 2016; Lu et al., 2015). One potential explanation is that QoL reflects a multidimensional construct influenced by physical health, psychological well-being, social participation, and environmental factors (The WHOQOL Group, 1998). Improvements in physical symptoms alone may therefore be insufficient to produce immediate changes in broader QoL measures. Age was the only participant characteristic that significantly moderated QoL outcomes. Younger patients (<60 years) experienced substantial improvements in QoL, whereas no significant effects were observed in older participants. This difference may be explained by several factors. Younger individuals may experience greater improvements in daily activity participation and social engagement following rehabilitation, which can positively influence perceived quality of life (Stubbs et al., 2013). Additionally, younger patients often have higher expectations regarding functional recovery and lifestyle participation, making improvements more noticeable and meaningful from a psychosocial perspective. In contrast, older adults may experience multiple health-related limitations and chronic conditions that influence overall well-being beyond the effects of knee rehabilitation alone (Cruz-Jentoft et al., 2019). Intervention characteristics showed less consistent moderating effects for QoL outcomes. Neither session length nor training frequency significantly influenced QoL improvements. However, the analysis suggested a potential difference related to total intervention duration, with longer programs showing a positive effect trend compared with shorter interventions. This observation may reflect the delayed nature of psychosocial adaptation during rehabilitation. Improvements in quality of life often occur gradually as patients regain confidence in movement, increase participation in daily activities, and experience sustained symptom relief. Intervention periods may be required for these broader psychosocial benefits to emerge. Another important consideration is the measurement of QoL outcomes. Most studies included in the present analysis assessed QoL using the KOOS QoL subscale, which primarily captures knee-related lifestyle limitations rather than broader psychosocial well-being (Collins et al., 2016). As a result, subtle improvements in emotional or social dimensions of recovery may not have been fully captured. Future studies incorporating multidimensional QoL instruments may provide a more comprehensive assessment of the psychosocial benefits of aquatic rehabilitation.

In the GRADE evaluation, the overall certainty of evidence for all primary outcomes was rated as very low, reflecting several methodological and statistical limitations inherent to the included trials. Most notably, all studies involved exercise-based rehabilitation, where blinding of participants and therapists is inherently infeasible. Accordingly, all trials were rated as having “some concerns” in the domain of deviations from intended interventions under the RoB 2.0 framework. This lack of blinding introduces a risk of performance bias, particularly for self-reported outcomes such as pain perception and QoL. While the present meta-analysis sought to mitigate these concerns by prioritizing objectively measured outcomes (e.g., muscle strength, proprioception, flexibility, balance, mobility) and incorporating data from studies with blinded assessors where available, the influence of participants’ expectations cannot be fully excluded. Another major contributor to the downgraded certainty was the substantial inconsistency observed across studies. Heterogeneity was high for symptoms and physical function, and remained considerable for QoL. Subgroup analyses based on comparator conditions and sensitivity testing were conducted to explore potential sources. However, substantial heterogeneity persisted, suggesting that variations in intervention protocols and participant profiles likely reflect genuine clinical diversity. Such variability reduces confidence in the precision and generalizability of the pooled estimates, underscoring the need for greater methodological standardization and clearer reporting of intervention parameters in future randomized trials. For QoL, the evidence was additionally downgraded for imprecision, as the pooled effect estimate crossed the line of no effect, indicating limited statistical power. Taken together, GRADE evaluation indicates that while the observed benefits of aquatic rehabilitation are consistent and clinically meaningful, the confidence in the magnitude of these effects remains limited. Strengthening this evidence base requires rigorously designed randomized controlled trials with adequate randomization, allocation concealment, blinded outcome assessment, and standardized intervention protocols. Future studies should employ motion analysis, isokinetic testing, and imaging-based assessment to provide more objective and reproducible outcome measures. Despite these methodological limitations, the overall direction and consistency of effects suggest that aquatic rehabilitation remains clinically relevant and warrants continued application in practice.

The findings of the present meta-analysis have several important clinical implications for the rehabilitation of individuals with knee joint dysfunction. First, the overall results suggest that aquatic rehabilitation represents a viable and effective therapeutic option for improving symptoms and physical function. The buoyant properties of water reduce joint loading while allowing active movement, enabling patients with pain or limited weight-bearing tolerance to engage in therapeutic exercise with lower mechanical stress on the knee joint. This characteristic makes aquatic exercise particularly suitable for individuals with degenerative joint conditions such as knee osteoarthritis, where excessive joint loading often limits participation in conventional land-based rehabilitation. Second, the subgroup analyses provide clinically relevant insights into patient selection and program design. The strongest evidence base in the present review was observed in populations with knee osteoarthritis (KOA), where aquatic rehabilitation demonstrated consistent improvements in both symptoms and functional outcomes. In contrast, the evidence for post-surgical populations—including individuals following TKR, ACLR, UKR, or APM—was comparatively limited. These subgroups were represented by fewer trials and smaller sample sizes, and therefore, conclusions regarding the effectiveness of aquatic rehabilitation in these populations should be interpreted cautiously. While aquatic exercise may still serve as a valuable adjunct during early rehabilitation phases, extrapolation of the present findings beyond KOA populations requires confirmation from additional high-quality randomized trials. Third, several intervention characteristics identified in the subgroup analyses may help guide clinical practice. Programs lasting at least eight weeks were associated with greater improvements in symptoms and physical function, suggesting that sustained training exposure is necessary to achieve meaningful therapeutic benefits. Additionally, shorter session durations (<60 minutes) appeared to produce larger functional improvements, potentially reflecting better adherence and reduced fatigue among patients with pain or limited endurance. These findings suggest that clinicians may consider implementing moderate-duration sessions delivered consistently over longer intervention periods when designing aquatic rehabilitation programs.

Several limitations of this meta-analysis should be acknowledged. First, substantial statistical heterogeneity was observed across several pooled analyses, particularly for symptom and physical function outcomes. Although subgroup analyses were conducted to explore potential sources of variability, considerable heterogeneity remained. This variability likely reflects differences in participant characteristics, disease severity, rehabilitation protocols, outcome measures, and intervention durations across studies. Consequently, the pooled effect estimates should be interpreted with caution, as they may represent averages across diverse clinical contexts rather than uniform treatment effects. Second, the evidence base was unevenly distributed across different knee pathologies. The strongest evidence was derived from studies involving individuals with KOA, whereas the number of trials involving TKR, ACLR, UKR, and APM populations was comparatively small. The limited sample sizes within these subgroups reduce statistical power and restrict the generalizability of the findings. Therefore, conclusions regarding the effectiveness of aquatic rehabilitation in post-surgical populations should be interpreted cautiously, and further studies are required before firm clinical recommendations can be made for these patient groups. Third, methodological limitations of the included studies may have influenced the certainty of evidence. As is common in exercise-based rehabilitation research, blinding of participants and therapists was not feasible in most trials, which may increase the risk of performance bias. In addition, several outcomes—particularly pain and quality of life—were based on self-reported measures, which may be influenced by participants’ expectations and subjective perceptions. Variations in intervention protocols, including differences in exercise intensity, program structure, and supervision levels, may also have contributed to heterogeneity in treatment effects. Fourth, the measurement of outcomes varied considerably across studies. Although validated instruments such as the KOOS were frequently used, different studies employed diverse functional assessments and quality-of-life measures, which may limit direct comparability between trials. Furthermore, the QoL outcomes included in this analysis primarily captured knee-specific limitations rather than broader health-related quality of life, potentially underestimating the psychosocial benefits of rehabilitation.

Future research should aim to address these limitations and strengthen the evidence base for aquatic rehabilitation in knee disorders. First, additional high-quality randomized controlled trials are needed in post-surgical populations, including individuals following TKR, ACLR, UKR, and APM. Larger sample sizes and standardized reporting of rehabilitation protocols will be essential to clarify whether aquatic rehabilitation provides comparable benefits in these clinical contexts. Second, future studies should adopt more standardized intervention protocols and clearly report key parameters such as water depth, exercise intensity, session duration, and training frequency. Greater methodological consistency would facilitate comparison across studies and help identify the most effective aquatic rehabilitation strategies. Third, the use of objective outcome measures should be expanded. Incorporating motion analysis, isokinetic strength testing, biomechanical assessments, and wearable activity monitoring could provide more precise and reproducible indicators of functional improvement. These objective measures may also help reduce potential bias associated with self-reported outcomes. Fourth, future trials should include comprehensive assessments of health-related quality of life using multidimensional instruments that capture psychological, social, and participation-related outcomes. Such approaches would provide a more complete understanding of the broader benefits of aquatic rehabilitation beyond symptom relief and functional recovery. Finally, long-term follow-up studies are needed to determine whether the benefits of aquatic rehabilitation are sustained over time and whether early improvements translate into lasting improvements in physical activity participation and joint health.

In conclusion, this systematic review and meta-analysis demonstrate that aquatic rehabilitation provides meaningful improvements in symptoms and physical function in individuals with knee joint dysfunction, particularly among patients with KOA. While improvements in quality of life were less consistent, the overall findings support aquatic exercise as a safe and clinically valuable rehabilitation strategy. Subgroup analyses suggest that patient characteristics and intervention parameters—such as disease type, age, and total intervention duration—may influence treatment effectiveness. However, the overall certainty of evidence remains limited due to methodological constraints, heterogeneity among studies, and the relatively small number of trials in certain patient populations. Further rigorously designed randomized controlled trials with standardized intervention protocols and comprehensive outcome assessment are needed to clarify the optimal role of aquatic rehabilitation across different knee disorders.

## Data Availability

The original contributions presented in the study are included in the article/**Supplementary Material**. Further inquiries can be directed to the corresponding author.
