# Supplementary material for: Effects of aquatic rehabilitation on symptoms, physical function, and quality of life in individuals with knee joint dysfunction: a meta-analysis
Source: Front Physiol. 2026 Apr 2;17:1804926. doi: 10.3389/fphys.2026.1804926 (PMC13082935; doi:10.3389/fphys.2026.1804926)
Supplement: Supplementary file 1 [file DataSheet1.docx]

Supplementary Material

Contents

[1 Search strategy 1](#_Toc3903)

[2 Characteristics of included studies 5](#_Toc17509)

[3 Forest plot of the overall pooled effects of aquatic rehabilitation compared with control conditions 10](#_Toc21494)

[4 Forest plots of subgroup analyses 13](#_Toc8294)

[5 Risk of bias assessment and publication bias analyses 39](#_Toc28955)

# Search strategy

All database searches were conducted on July 14, 2025.

**PubMed:**

**Set 1:** ((((((((((knee[Title/Abstract]) OR (ligament[Title/Abstract])) OR (menisc*[Title/Abstract])) OR (cartilage[Title/Abstract])) OR (chondr*[Title/Abstract])) OR (osteochondral[Title/Abstract])) OR (tibia*[Title/Abstract])) OR (tibio*[Title/Abstract])) OR (femur[Title/Abstract])) OR (femor*[Title/Abstract])) OR (patell*[Title/Abstract])

**Set 2:** (((((((((((hydrotherapy[Title/Abstract]) OR (aquatic*[Title/Abstract])) OR (aquatic exercise[Title/Abstract])) OR (water exercise[Title/Abstract])) OR (aquatic rehabilitation[Title/Abstract])) OR (water rehabilitation[Title/Abstract])) OR (aquatic therapy[Title/Abstract])) OR (water therapy[Title/Abstract])) OR (hot tub[Title/Abstract])) OR (balneology[Title/Abstract])) OR (bath*[Title/Abstract])) OR (swim*[Title/Abstract])) OR (therapeutic irrigation[Title/Abstract])

**Set 3:** randomized controlled trial*[Title/Abstract]

**Final search strategy:** #1 AND #2 AND #3

**Results:** 97 records

**Web of Science (Core Collection):**

**Set 1:** ((((((((((TS=(knee)) OR TS=(ligament)) OR TS=(menisc*)) OR TS=(cartilage)) OR TS=(chondr*)) OR TS=(osteochondral)) OR TS=(tibia*)) OR TS=(tibio*)) OR TS=(femur)) OR TS=(femor*)) OR TS=(patell*)

**Set 2:** (((((((((((TS=(hydrotherapy)) OR TS=(aquatic*)) OR TS=(aquatic exercise)) OR TS=(water exercise)) OR TS=(aquatic rehabilitation)) OR TS=(water rehabilitation)) OR TS=(aquatic therapy)) OR TS=(water therapy)) OR TS=(hot tub)) OR TS=(balneology)) OR TS=(bath*)) OR TS=(swim*)) OR TS=(therapeutic irrigation)

**Set 3:** TS=(randomized controlled trial*)

**Final search strategy:** #1 AND #2 AND #3

**Results:** 370 records

**Cochrane Library:**

**Set 1:** (knee):ti,ab,kw OR (ligament):ti,ab,kw OR (menisc*):ti,ab,kw OR (cartilage):ti,ab,kw OR (chondr*):ti,ab,kw OR (osteochondral):ti,ab,kw OR (tibia*):ti,ab,kw OR (tibio*):ti,ab,kw OR (femur):ti,ab,kw OR (femor*):ti,ab,kw OR (patell*):ti,ab,kw

**Set 2:** (hydrotherapy): ti, ab, kw OR (aquatic*): ti, ab, kw OR (aquatic exercise): ti, ab, kw OR (water exercise): ti, ab, kw OR (aquatic rehabilitation): ti, ab, kw OR (water rehabilitation): ti, ab, kw OR (aquatic therapy): ti, ab, kw OR (water therapy): ti, ab, kw OR (hot tub): ti, ab, kw OR (balneology): ti, ab, kw OR (bath*): ti, ab, kw OR (swim*): ti, ab, kw OR (therapeutic irrigation): ti, ab, kw

**Set 3:** (randomized controlled trial*): ti, ab, kw

**Final search strategy:** #1 AND #2 AND #3

**Results:** 552 records (20 from Cochrane Reviews; 532 from Trials)

**SPORTDiscus:**

**Search strategy:** ((knee) OR (ligament) OR (menisc*) OR (cartilage) OR (chondr*) OR (osteochondral) OR (tibia*) OR (tibio*) OR (femur) OR (femor*) OR (patell*)) AND ((hydrotherapy) OR (aquatic*) OR (aquatic exercise) OR (water exercise) OR (aquatic rehabilitation) OR (water rehabilitation) OR (aquatic therapy) OR (water therapy) OR (hot tub) OR (balneology) OR (bath*) OR (swim*) OR (therapeutic irrigation)) AND (randomized controlled trial*)

**Results:** 74 records

**CINAHL:**

**Search strategy:** (knee OR ligament OR menisc* OR cartilage OR chondr* OR osteochondral OR tibia* OR tibio* OR femur OR femor* OR patell*) AND (hydrotherapy OR aquatic* OR aquatic exercise OR water exercise OR aquatic rehabilitation OR water rehabilitation OR aquatic therapy OR water therapy OR hot tub OR balneology OR bath* OR swim* OR therapeutic irrigation) AND (randomized controlled trial*)

**Results:** 162 records

**Embase:**

**Set 1:** 'knee':ab,ti OR 'ligament':ab,ti OR 'menisc*':ab,ti OR 'cartilage':ab,ti OR 'chondr*':ab,ti OR 'osteochondral':ab,ti OR 'tibia*':ab,ti OR 'tibio*':ab,ti OR 'femur':ab,ti OR 'femor*':ab,ti OR 'patell*':ab,ti

**Set 2:** 'hydrotherapy':ab,ti OR 'aquatic*':ab,ti OR 'aquatic exercise':ab,ti OR 'water exercise':ab,ti OR 'aquatic rehabilitation':ab,ti OR 'water rehabilitation':ab,ti OR 'aquatic therapy':ab,ti OR 'water therapy':ab,ti OR 'hot tub':ab,ti OR 'balneology':ab,ti OR 'bath*':ab,ti OR 'swim*':ab,ti OR 'therapeutic irrigation':ab,ti

**Set 3:** 'randomized controlled trial*':ab, ti

**Final search strategy:** #1 AND #2 AND #3

**Results:** 136

# Characteristics of included studies

**Supplementary 2 Table S1.** Characteristics of included studies

| Study | Country | Sample  (Female%) | Population | Age | Intervention protocol | | | | | Comparison |
| --- | --- | --- | --- | --- | --- | --- | --- | --- | --- | --- |
|  |  |  |  |  | Session length  Frequency | Duration | Type of exercise | Water depth | WT |  |
| Assar 2020 | Iran | 36  (100%) | Female with KOA | 59.1 (8.2) | 90 min  3 times/wk | 8 weeks | Stretching, Aerobic, Strength, Resistance, Proprioception, Step | 1.3 m | 32°C | LBG: TRE  CTG: drug regimens |
| Azizi 2020 | Iran | 32  (0%) | Elderly male with KOA | 64.5 (4.1) | 60 min  3 times/wk | 8 weeks | Strength, Resistance | 1.2 m | 32°C | CTG: drug regimens and followed lifestyle |
| Dias 2017 | Brazil | 65  (100%) | Elderly female with KOA | 70.8 (5.1) | 40min  2 times/wk | 6 weeks | Progressive strength, Resistance | Supra-umbilical | 32°C | EG: educational intervention |
| Hajouj 2021 | Iran | 38  (0%) | Male athletes with ACLR | 24.2 (3.4) | 45-60 min  2 times/wk | 6 weeks | Stretching, Resistance,  Proprioception | Waist level | 28-30°C | LBG: multimodal exercise program |
| Harmer 2009 | Australia | 102  (56.9%) | Patients after TKR | 68.3 (7.9) | 60 min  2 times/wk | 6 weeks | Strength, Aerobic, Step, Resistance | Waist level | 25±3°C | LBG: multimodal exercise program |
| Jain 2025 | India | 290  (88.3%) | Patients with bilateral KOA | 54.9 (10.8) | 40-50 min  3 times/wk | 8 weeks | Strength, Resistance, Balance, Proprioception | Xiphoid level | 36°C | LBG: multimodal exercise program |
| Kalkhoran 2025 | Iran | 34  (100%) | Elderly female with KOA | 65.1 (1.5) | 60 min  2 times/wk | 8 weeks | Strength, Aerobic, Step, Resistance | Chest level | 34-36°C | CTG: usual care |
| Khruakhorn 2021 | Thailand | 34  (91.2%) | Patients with KOA | 61.4 (8.3) | 45-60 min  3 times/wk | 6 weeks | Progressive strength, Stretching, Resistance | NR | 32-33°C | LBG: multimodal exercise program |
| Krishnan 2021 | Malaysia | 45  (51.1%) | Obese patients  with KOA | 50.3 (5.3) | 60 min  2 times/wk | 8 weeks | Stretching, Strength, Aerobic, Resistance, Proprioception, Step | 1.2 m | 36°C | LBG1: elastic band exercise  LBG2: home exercise |
| Kuptniratsaikul 2019 | Thailand | 80  (93.8%) | Obese patients  with KOA | 61.9 (6.6) | 30 min  3 times/wk | 4 weeks | Strength, Aerobic, Resistance | NR | NR | LBG: home exercise |
| Li 2022 | China | 57  (40.4%) | Patients following ACLR | 33.4 (12.0) | 30 min  4 times/wk | 3 weeks | Aerobic, Resistance | Xiphoid level | 34.5°C | LBG: overground treadmill walking |
| Lim 2010 | Korea | 75  (86.7%) | Obese patients  with KOA | 65.6 (7.6) | 40 min  3 times/wk | 8 weeks | Stretching, Strength, Aerobic, Resistance, Endurance, Step | 1.15 m | 34°C | LBG1:multimodal exercise program  LBG2: home exercise |
| Lund 2008 | Denmark | 79  (78.5%) | Patients with KOA | 67.7 (10.9) | 50 min  2 times/wk | 8 weeks | Stretching, Strength, Endurance, Balance, Resistance | NR | 33.5°C | LBG: multimodal exercise program  NTG: no exercise |
| Munukka 2016 | Finland | 87  (100%) | Post-menopausal female, mild KOA | 64 (2) | 60 min  3 times/wk | 16 weeks | Progressive resistance | NR | 30-32°C | CTG: usual care |
| Munukka 2020 | Finland | 87  (100%) | Post-menopausal female, mild KOA | 64 (2) | 60 min  3 times/wk | 16 weeks | Progressive resistance | NR | 30-32°C | CTG: usual care |
| Pipino 2023 | Switzerland | 24  (45.8%) | Patients following ACLR | 27.2 (NR) | NR  3 times/wk | 4 weeks | Strength, Aerobic, Resistance, Proprioception | NR | 32°C | LBG: multimodal exercise program |
| Rewald 2020 | Netherlands | 102  (61.8%) | Patients with unilateral KOA | 59.9 (8.6) | 45 min  2 times/wk | 12 weeks | Aerobic, Resistance | Upper thoracic | 32°C | CTG: usual care |
| Sandeep 2025 | India | 150  (100%) | Obese female with bilateral KOA | 51.4 (2.7) | 55-60 min  3 times/wk | 8 weeks | Stretching, Strength, Resistance, Aerobic, Balance, Proprioception | 1.2 m | 36°C | LBG: multimodal exercise program |
| Silva 2008 | Brazil | 64  (92.2%) | Patients with KOA | 59 (6.9) | 50 min  3 times/wk | 18 weeks | Stretching, Strength, Resistance, Step | 1.2 m | 32°C | LBG: multimodal exercise program |
| Slouma 2024 | Tunisia | 60  (76.7%) | Patients with KOA | 54.9 (9.5) | 60 min  3 times/wk | 8 weeks | Strength, Endurance, Resistance, Proprioception | Xiphoid level | 26°C | LBG: multimodal exercise program |
| Taglietti 2018 | Brazil | 60  (68.3%) | Patients with KOA | 68.3 (4.8) | 60 min  2 times/wk | 8 weeks | Stretching, Strength, Aerobic, Step, Resistance, Proprioception | 1.2 m | 32°C | EG: educational intervention |
| Tamin 2018 | Indonesia | 33  NR | Obese patients  with KOA | 62.9 (45.4) | NR  3 times/wk | 8 weeks | Progressive aerobic, Strength, Resistance | 1.2 m | 32°C | LBG: multimodal exercise program |
| Valtonen 2010 | Finland | 50  (60.0%) | Patients after UKR | 66.0 (6.1) | 43-53min  2 times/wk | 12 weeks | Strength, Resistance | NR | NR | NTG: no intervention |
| Waller 2017 | Finland | 87  (100%) | Post-menopausal female, mild KOA | 63.9 (2.4) | 60 min  3 times/wk | 16 weeks | High intensity resistance | NR | NR | CTG: usual care |
| Wang 2011 | Taiwan | 78  (85.9%) | Patients with KOA | 67.7 (5.9) | 60 min  3 times/wk | 12 weeks | Flexibility, Aerobic, Resistance | NR | 30°C | LBG: multimodal exercise program  NTG: no intervention |
| Wyatt 2001 | America | 42  NR | Patients with KOA | 45-70 | NR  3 times/wk | 6 weeks | Strength, Aerobic, Resistance | 5 feet | 32°C | LBG: multimodal exercise program |
| Yennan 2010 | Thailand | 50  (100%) | Elderly female with  KOA | 66.0 (4.6) | 65 min  3 times/wk | 6 weeks | Stretching, Strength, Aerobic, Resistance, Proprioception | Waist level | Ambient | LBG: home exercise |
| Yesil 2023 | Turkey | 30  (66.7%) | Middle-aged active patients after APM | 45.15 (5.5) | 60 min  3 times/wk | 4 weeks | Stretching, Strength, Aerobic, Resistance | NR | 33°C | LBG: home exercise |
| Zamarioli 2008 | Brazil | 13  (7.7%) | Patients following ACLR | 18-55 | 50 min  2 times/wk | 9 weeks | Stretching, Strength, Resistance | NR | 33-34°C | LBG: multimodal exercise program |

Abbreviations: ACLR anterior cruciate ligament reconstruction, APM arthroscopic partial meniscectomy, CTG conventional treatment group, EG education group, Female% percentage of female, KOA knee osteoarthritis, LBG land-based training group, NR not reported, NTG no treatment group, TKR total knee replacement, TRE total resistance exercise, UKR unilateral knee replacement, WBG water-based training group, WT water temperature.

# **Forest plot of the overall pooled effects of aquatic rehabilitation compared with control conditions**

##
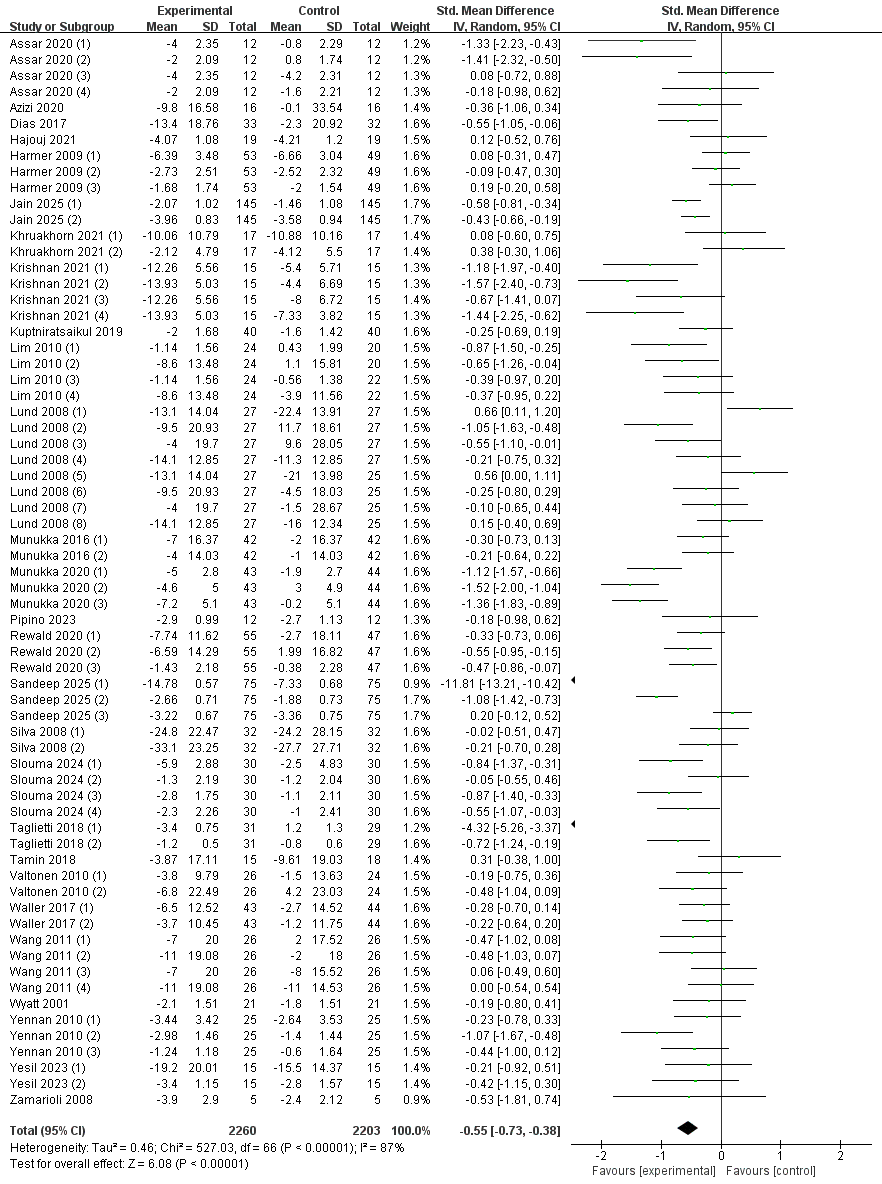


**Supplementary 3 Figure S1.** Forest plot of the overall pooled effects of aquatic rehabilitation compared with control conditions on symptom outcomes


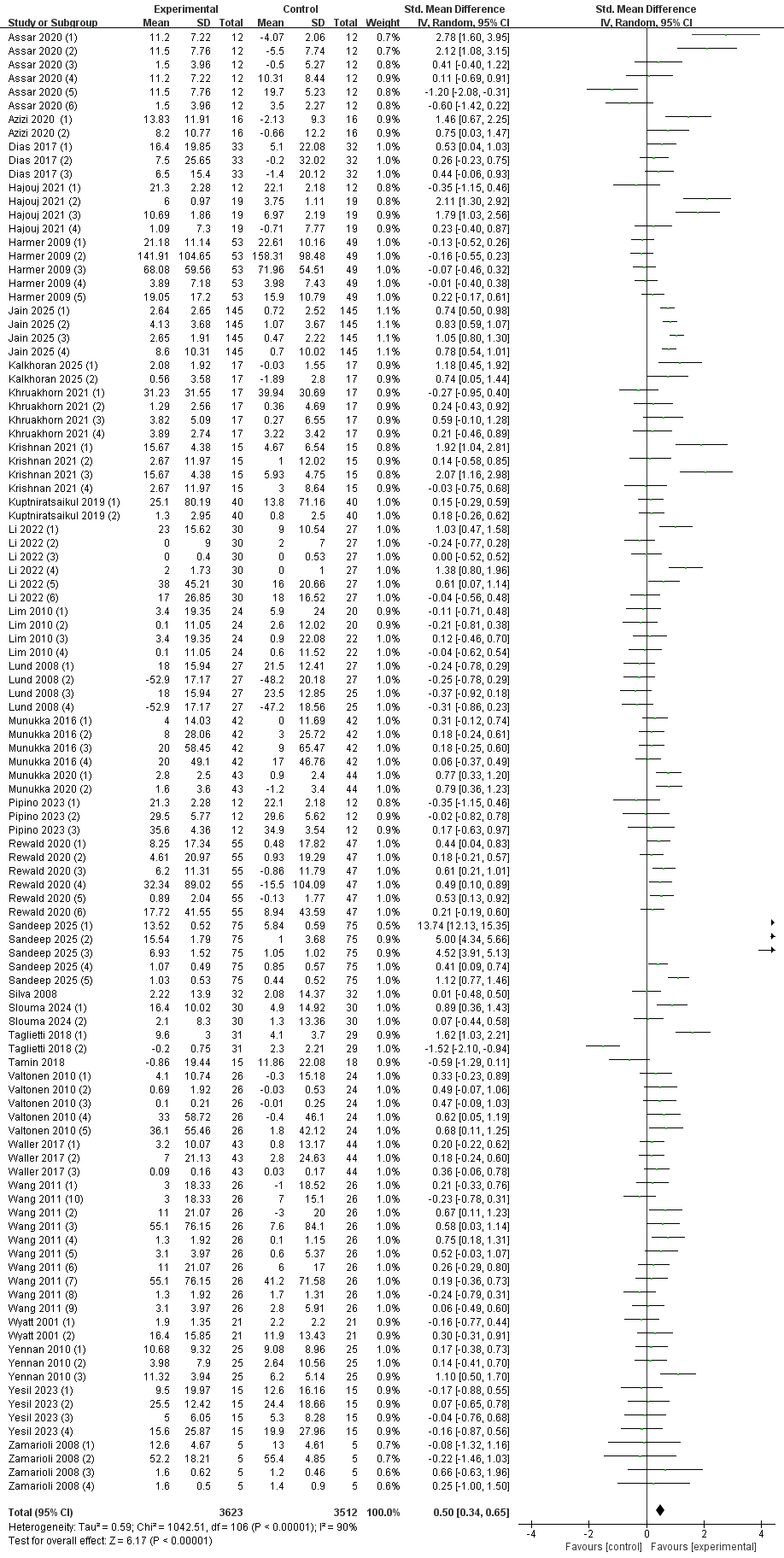


**Supplementary 3 Figure S2.** Forest plot of the overall pooled effects of aquatic rehabilitation compared with control conditions on physical function outcomes


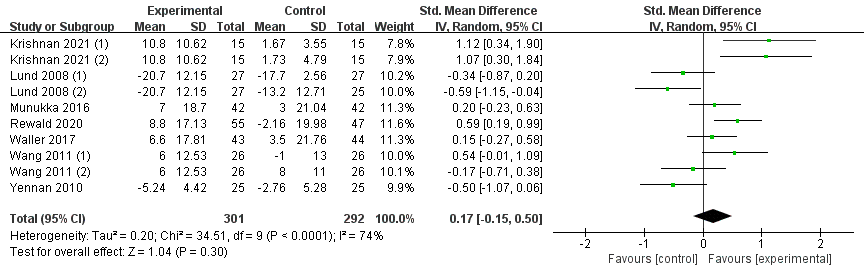


**Supplementary 3 Figure S3.** Forest plot of the overall pooled effects of aquatic rehabilitation compared with control conditions on quality of life outcomes

# Forest plots of subgroup analyses


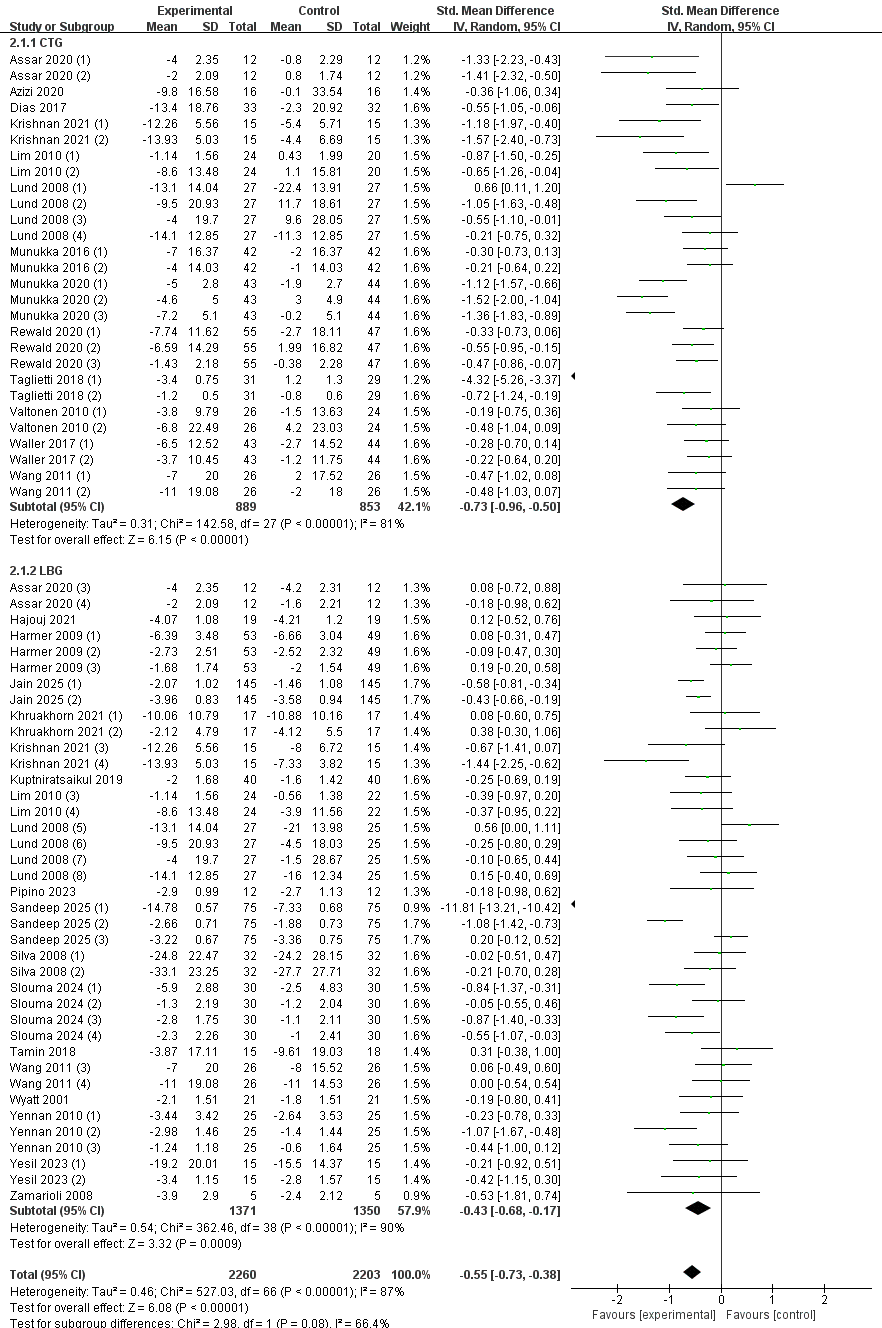


**Supplementary 4 Figure S4.** Forest plot of subgroup analyses examining the effects of aquatic rehabilitation versus control interventions on symptom outcomes, stratified by control type

Legend: CTG conventional treatment group, LBG land-based training group.


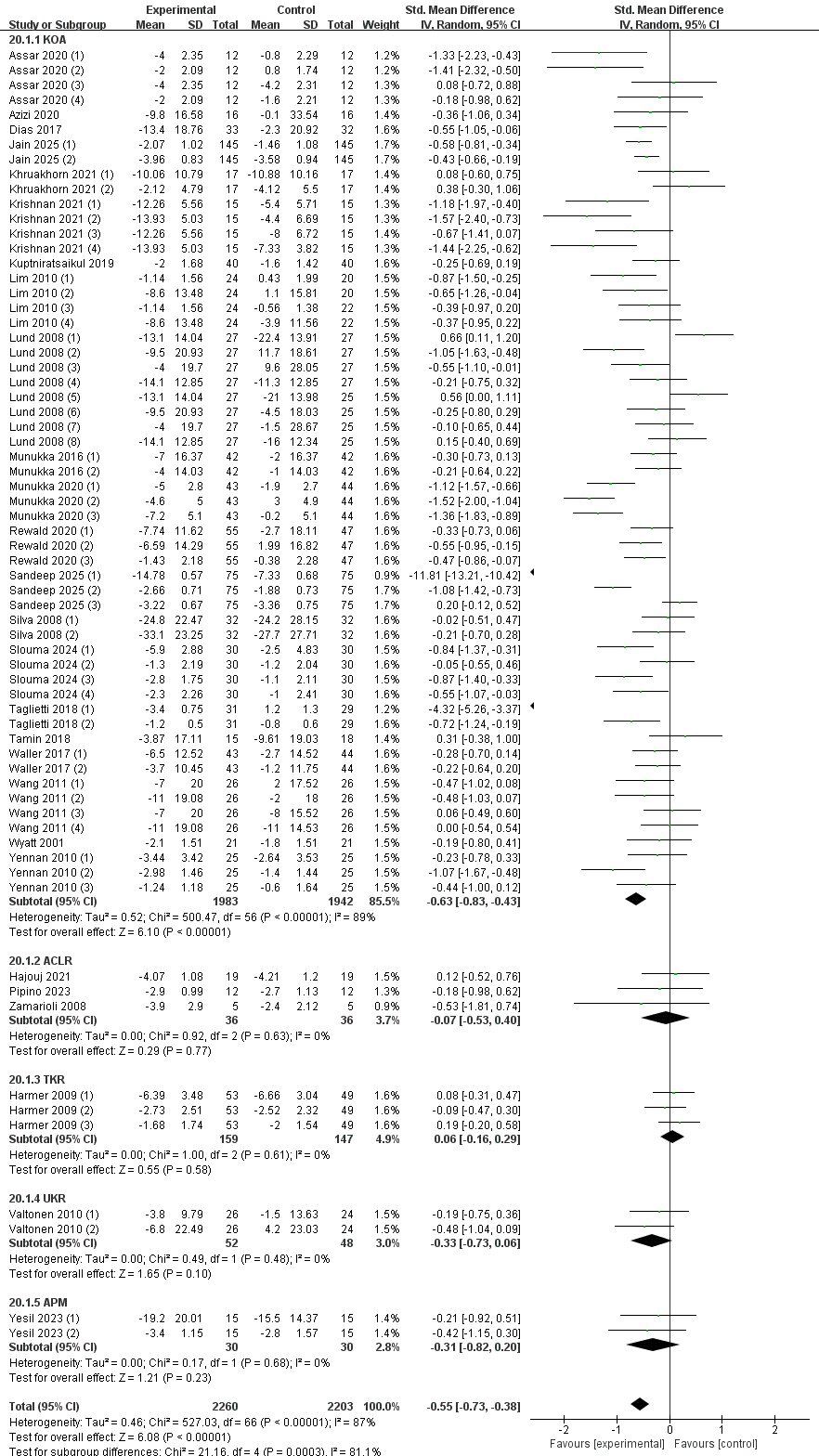


**Supplementary 4 Figure S5.** Forest plot of subgroup analyses examining the effects of aquatic rehabilitation versus control interventions on symptom outcomes, stratified by disease type

Legend: KOA knee osteoarthritis, ACLR anterior cruciate ligament reconstruction, TKR total knee replacement, UKR unilateral knee replacement, APM arthroscopic partial meniscectomy.


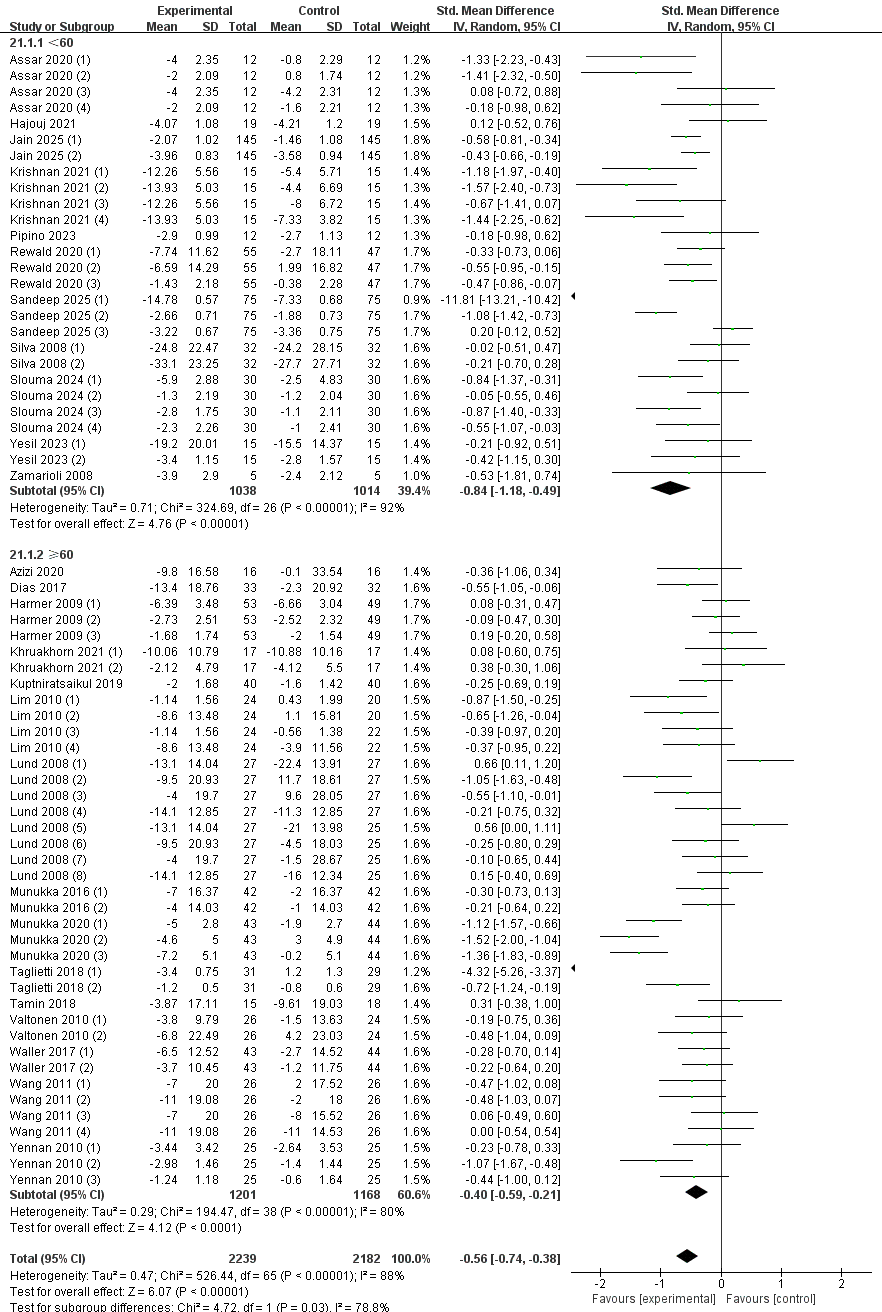


**Supplementary 4 Figure S6.** Forest plot of subgroup analyses examining the effects of aquatic rehabilitation versus control interventions on symptom outcomes, stratified by mean age


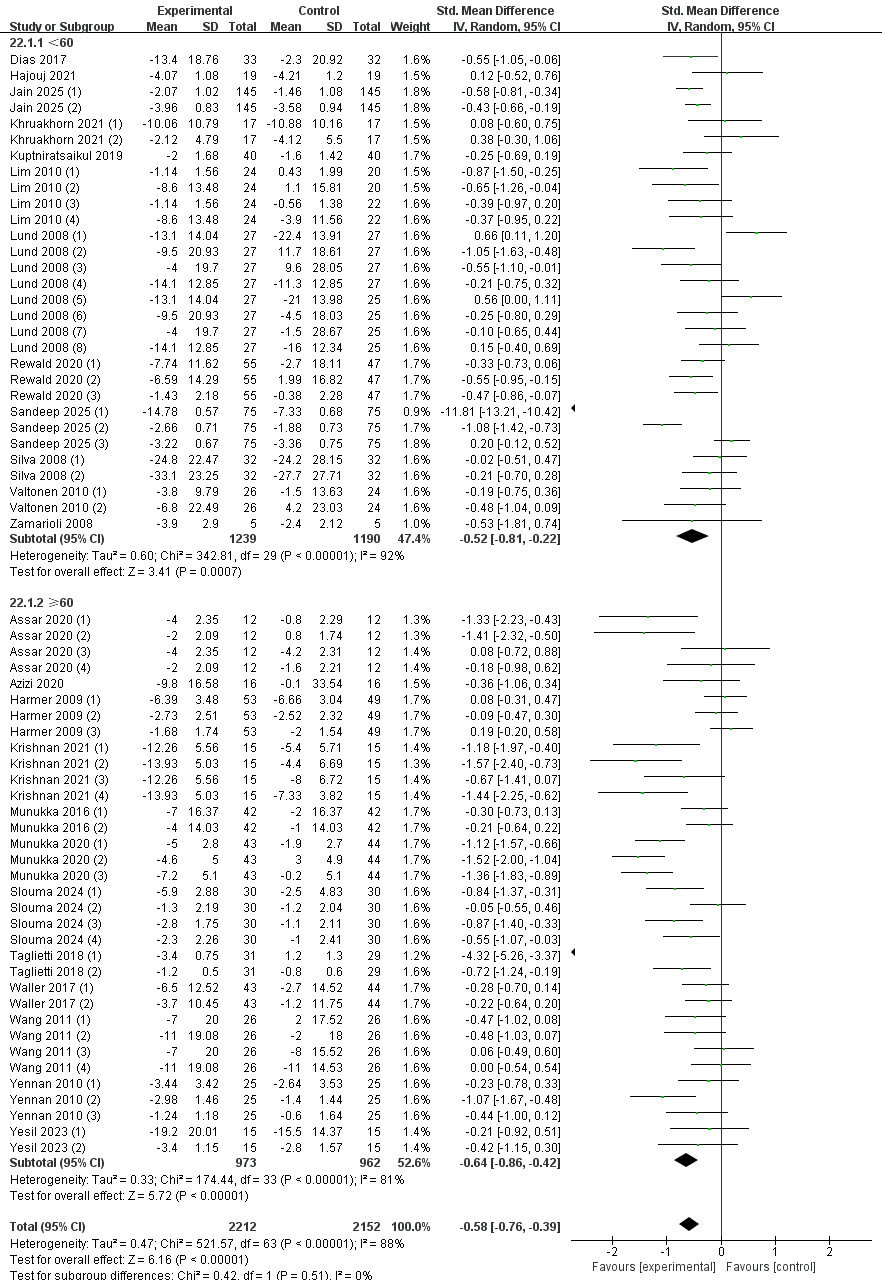


**Supplementary 4 Figure S7.** Forest plot of subgroup analyses examining the effects of aquatic rehabilitation versus control interventions on symptom outcomes, stratified by session length


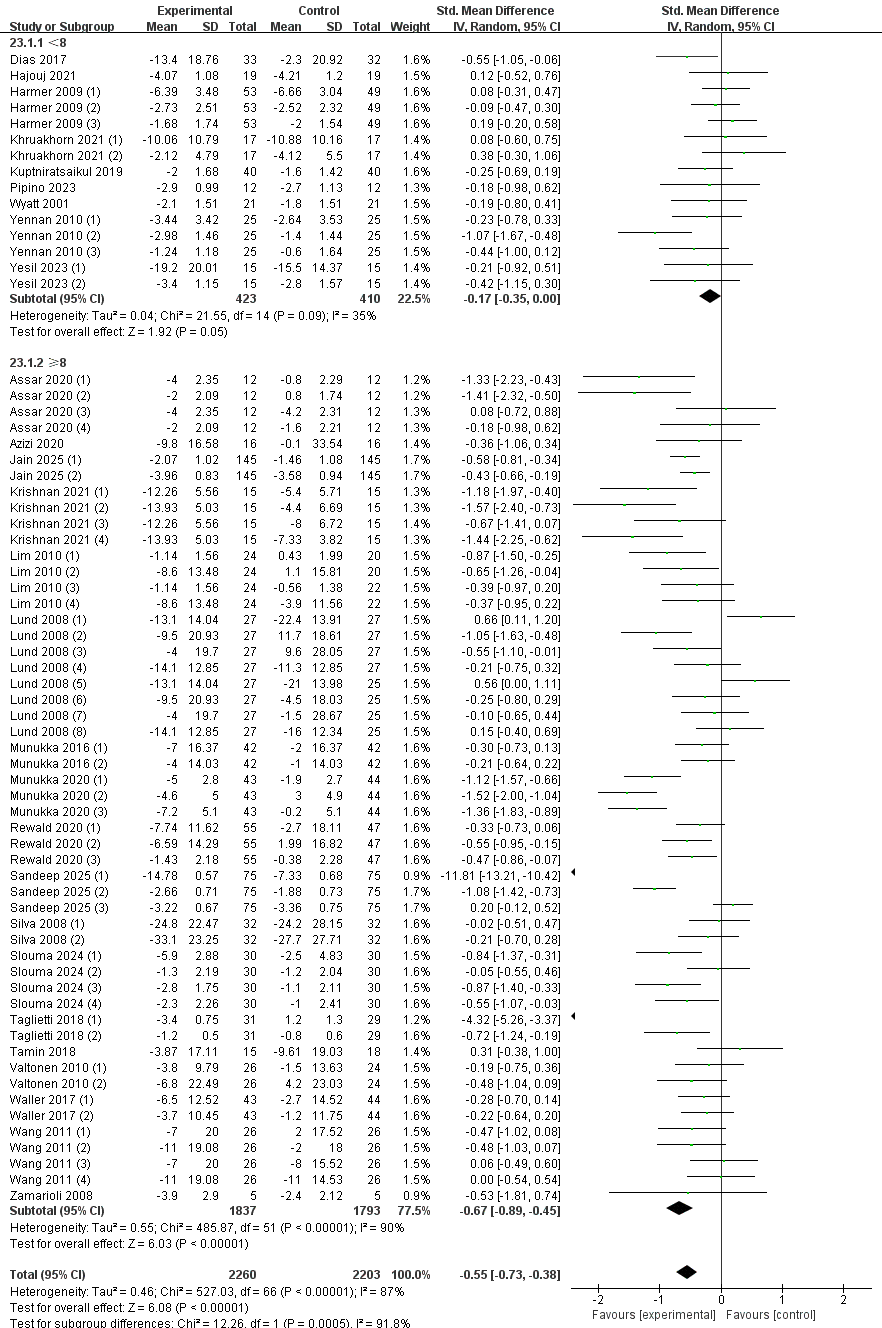


**Supplementary 4 Figure S8.** Forest plot of subgroup analyses examining the effects of aquatic rehabilitation versus control interventions on symptom outcomes, stratified by duration


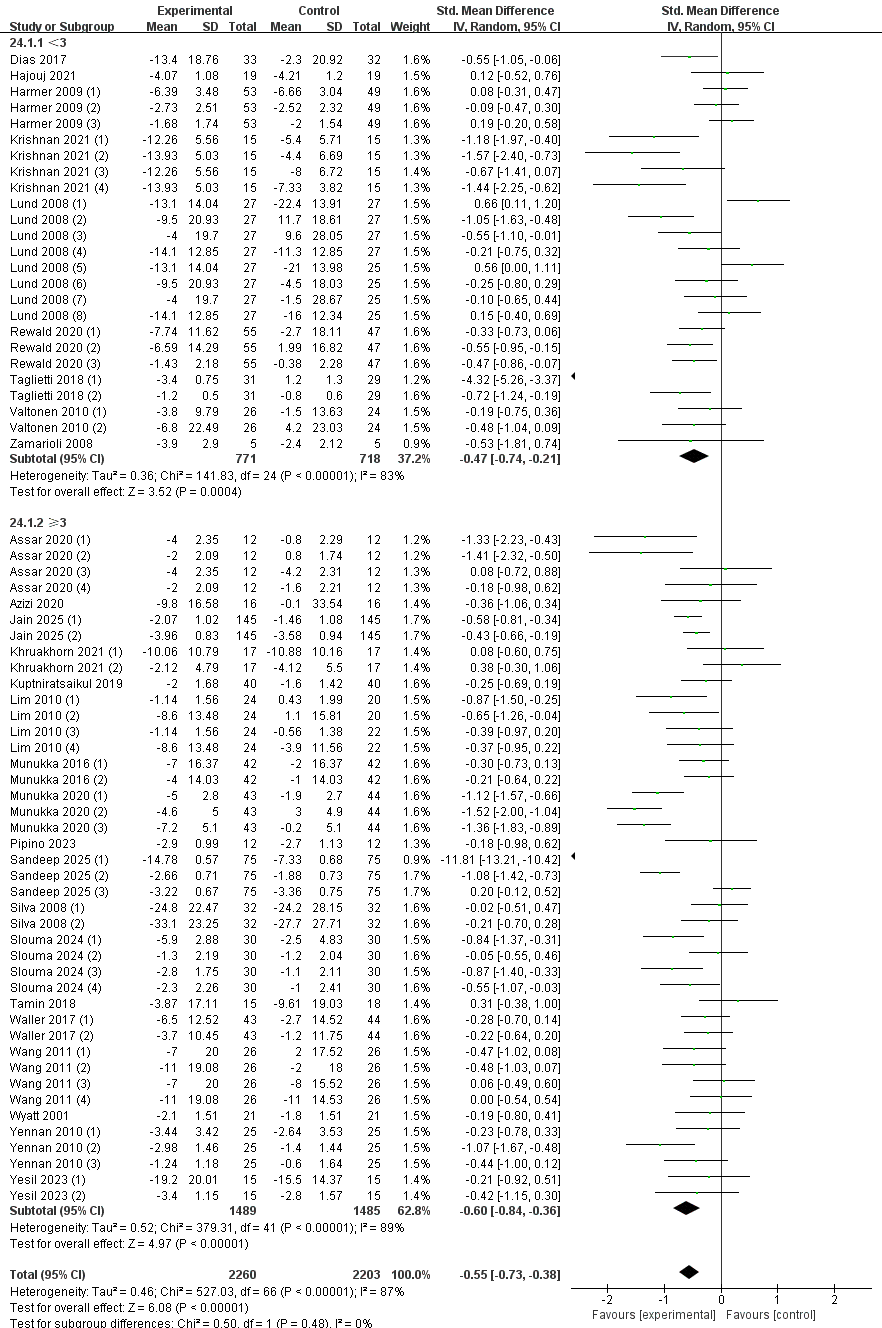


**Supplementary 4 Figure S9.** Forest plot of subgroup analyses examining the effects of aquatic rehabilitation versus control interventions on symptom outcomes, stratified by frequency


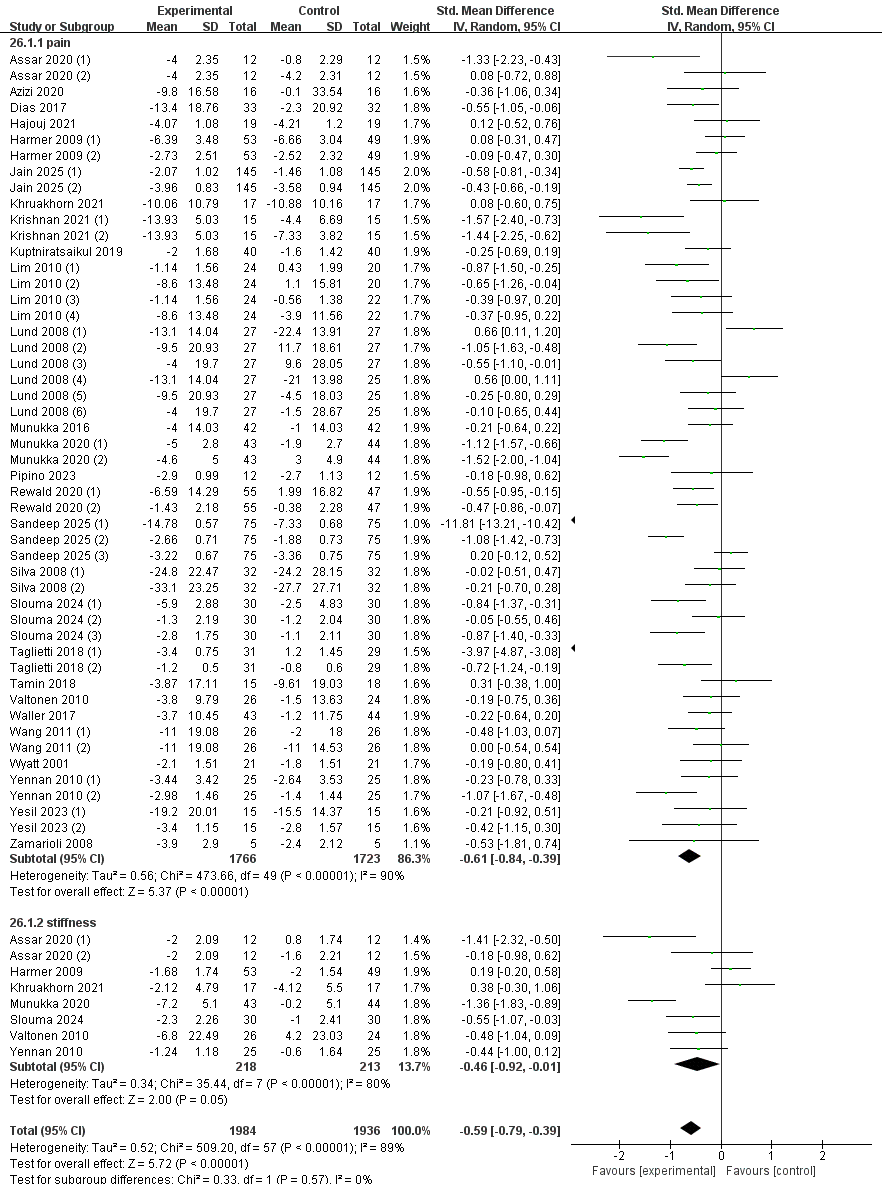


**Supplementary 4 Figure S10.** Forest plot of subgroup analyses examining the effects of aquatic rehabilitation versus control interventions on symptom outcomes, stratified by type of symptoms


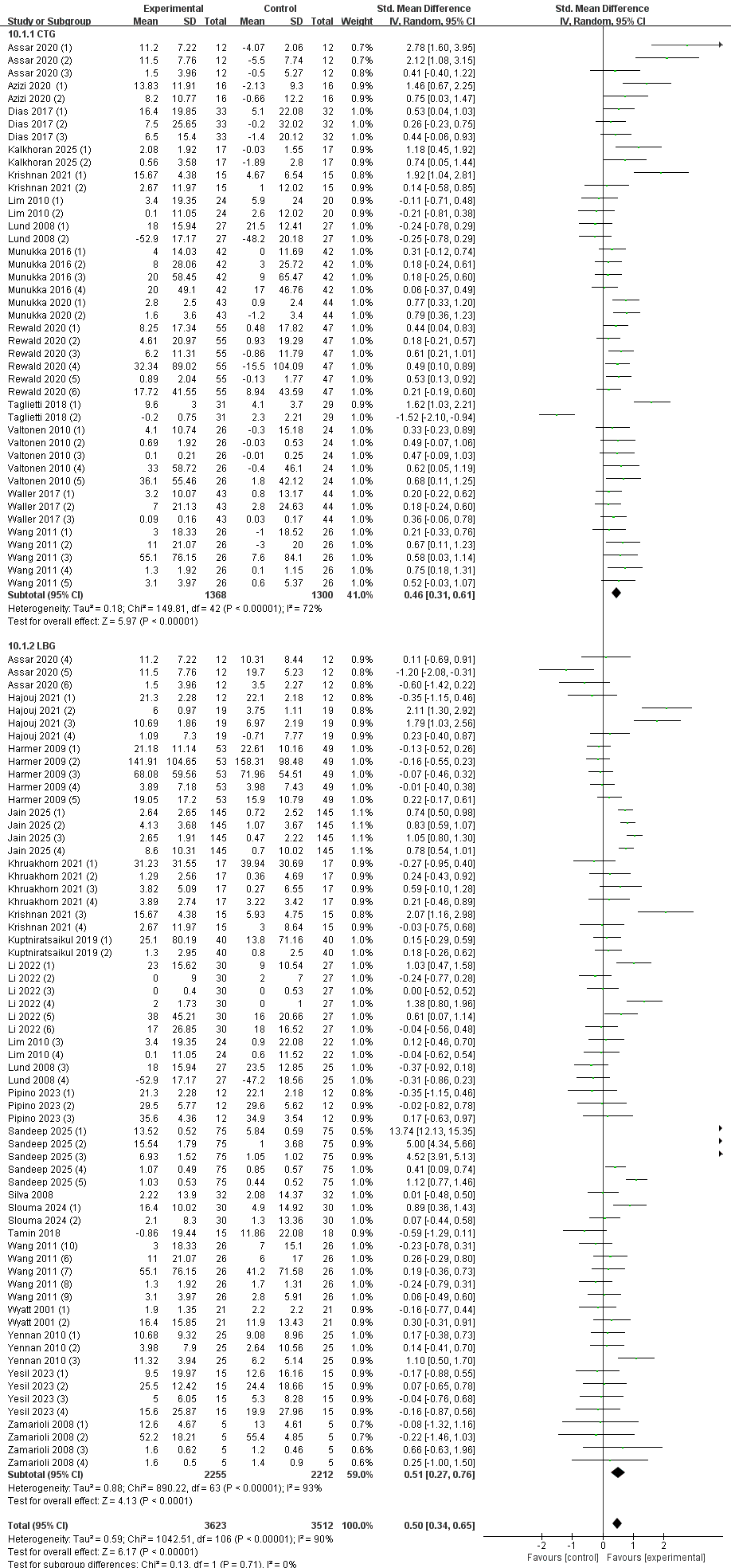


**Supplementary 4 Figure S11.** Forest plot of subgroup analyses examining the effects of aquatic rehabilitation versus control interventions on physical function, stratified by control type

Legend: CTG conventional treatment group, LBG land-based training group.


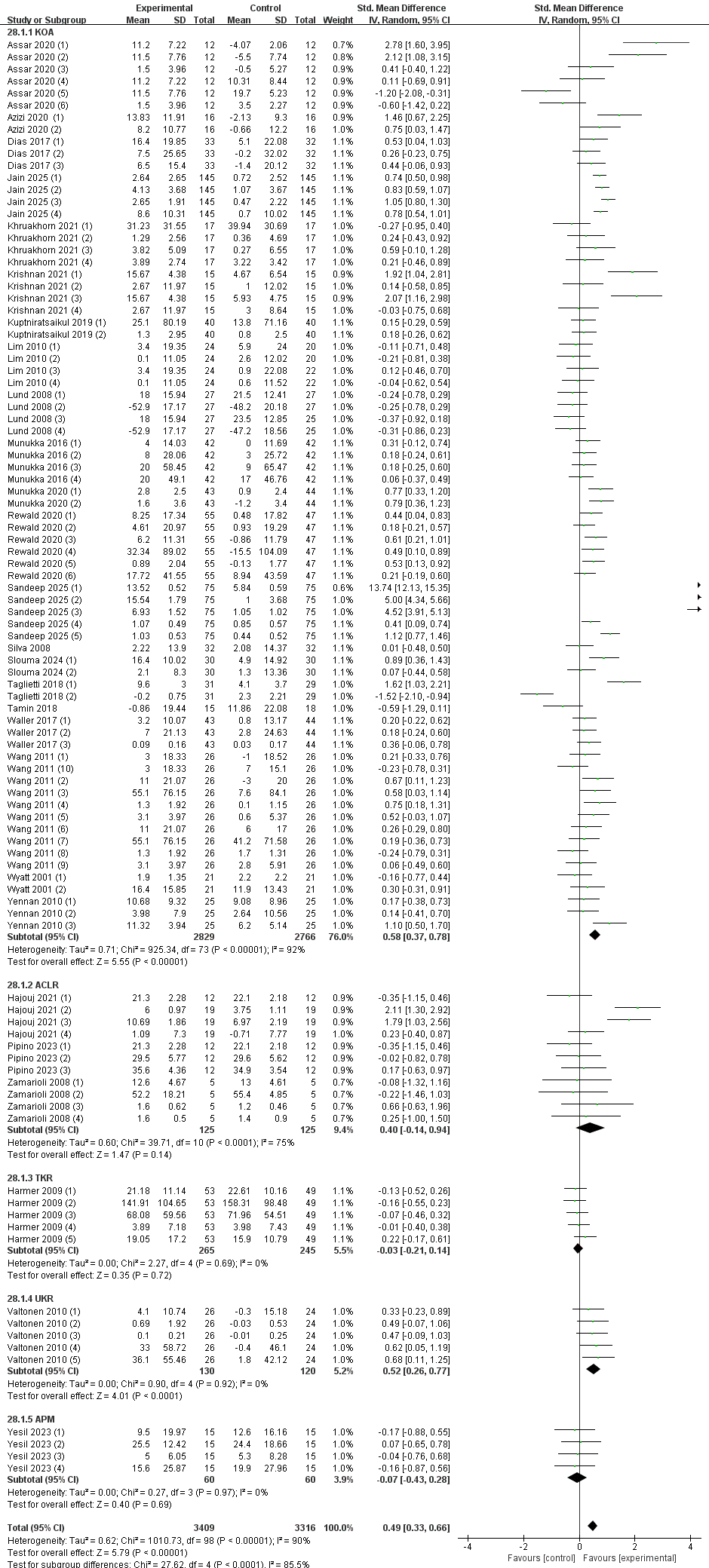


**Supplementary 4 Figure S12.** Forest plot of subgroup analyses examining the effects of aquatic rehabilitation versus control interventions on physical function, stratified by disease type

Legend: KOA knee osteoarthritis, ACLR anterior cruciate ligament reconstruction, TKR total knee replacement, UKR unilateral knee replacement, APM arthroscopic partial meniscectomy.


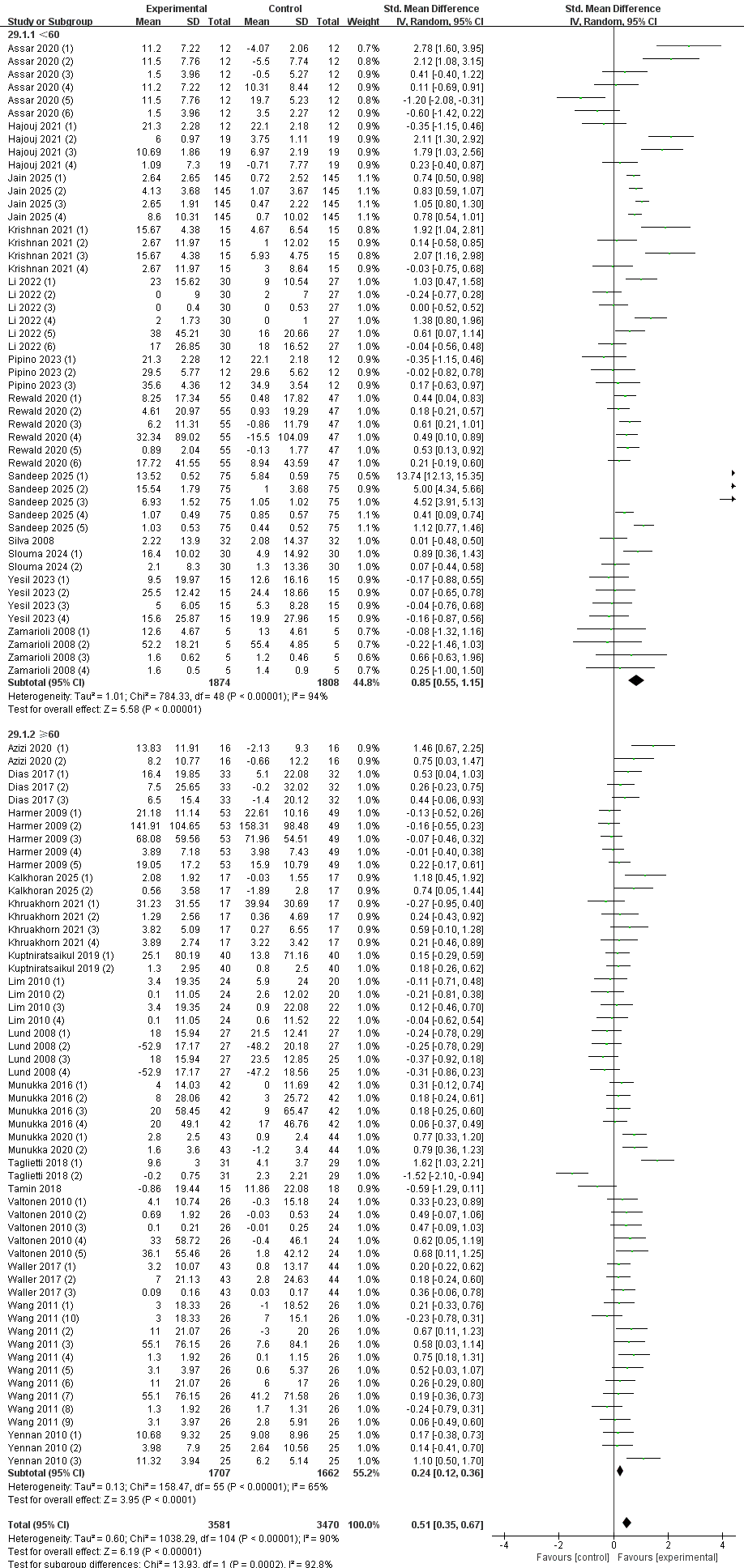


**Supplementary 4 Figure S13.** Forest plot of subgroup analyses examining the effects of aquatic rehabilitation versus control interventions on physical function, stratified by mean age


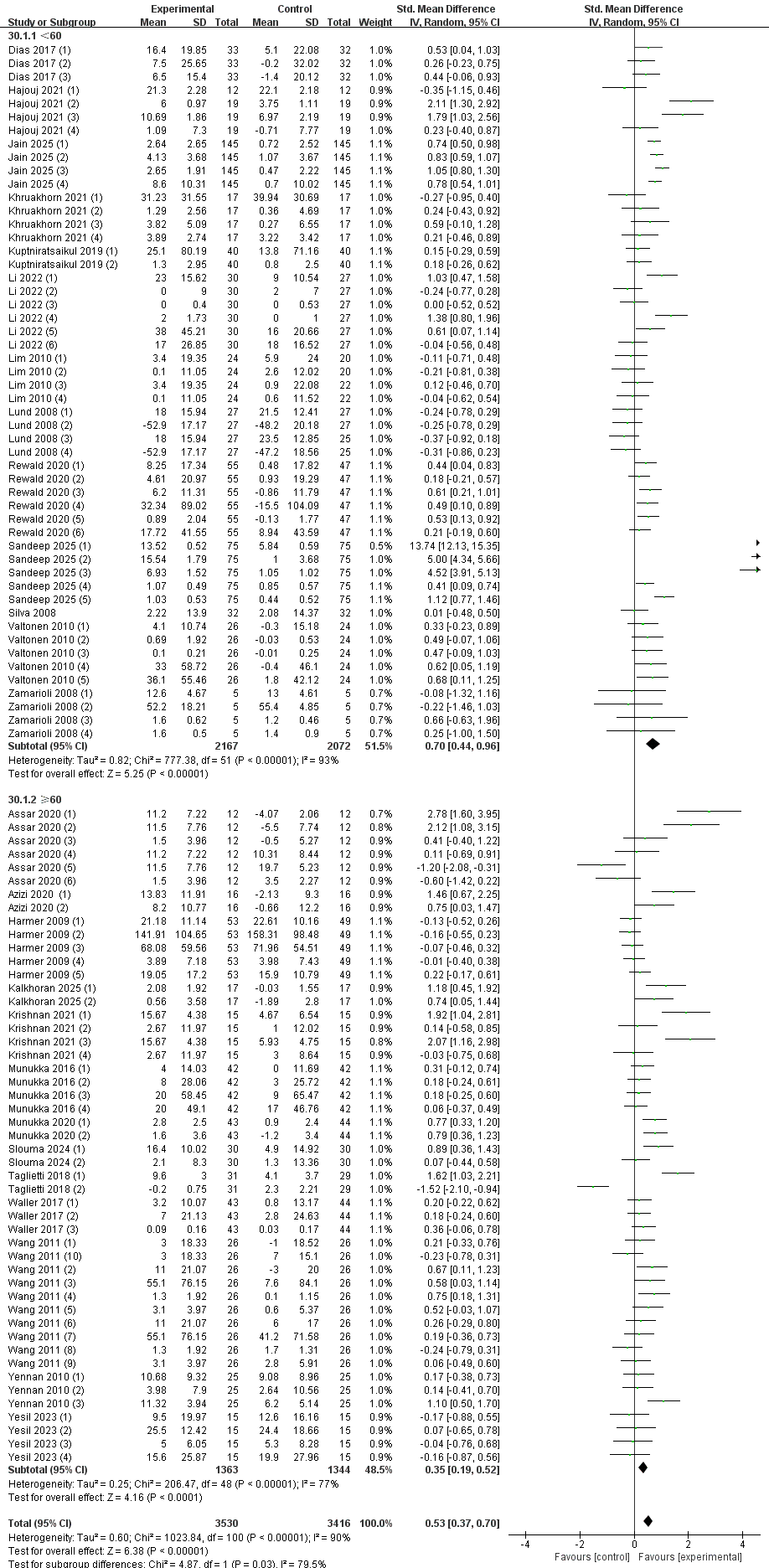


**Supplementary 4 Figure S14.** Forest plot of subgroup analyses examining the effects of aquatic rehabilitation versus control interventions on physical function, stratified by session length


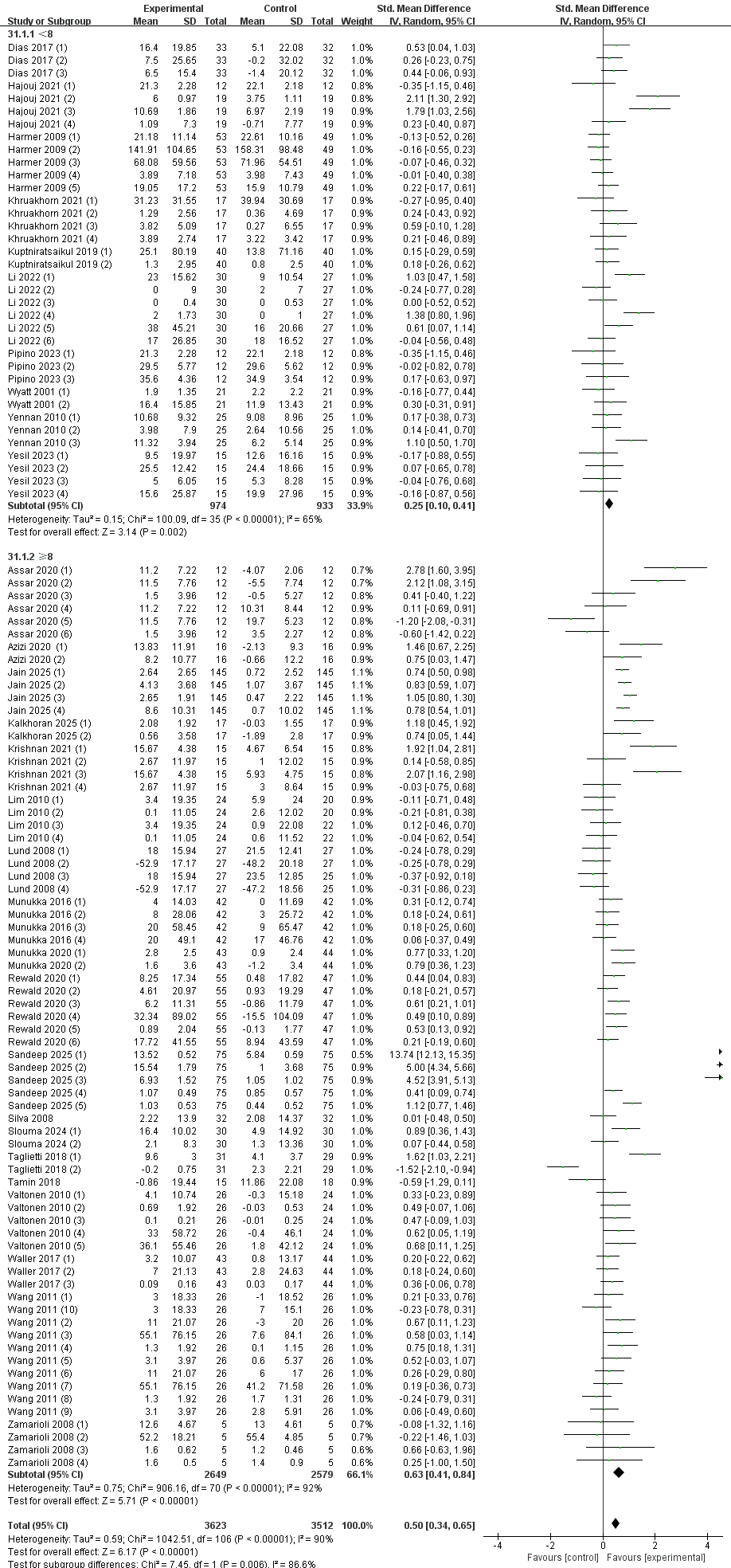


**Supplementary 4 Figure S15.** Forest plot of subgroup analyses examining the effects of aquatic rehabilitation versus control interventions on physical function, stratified by duration


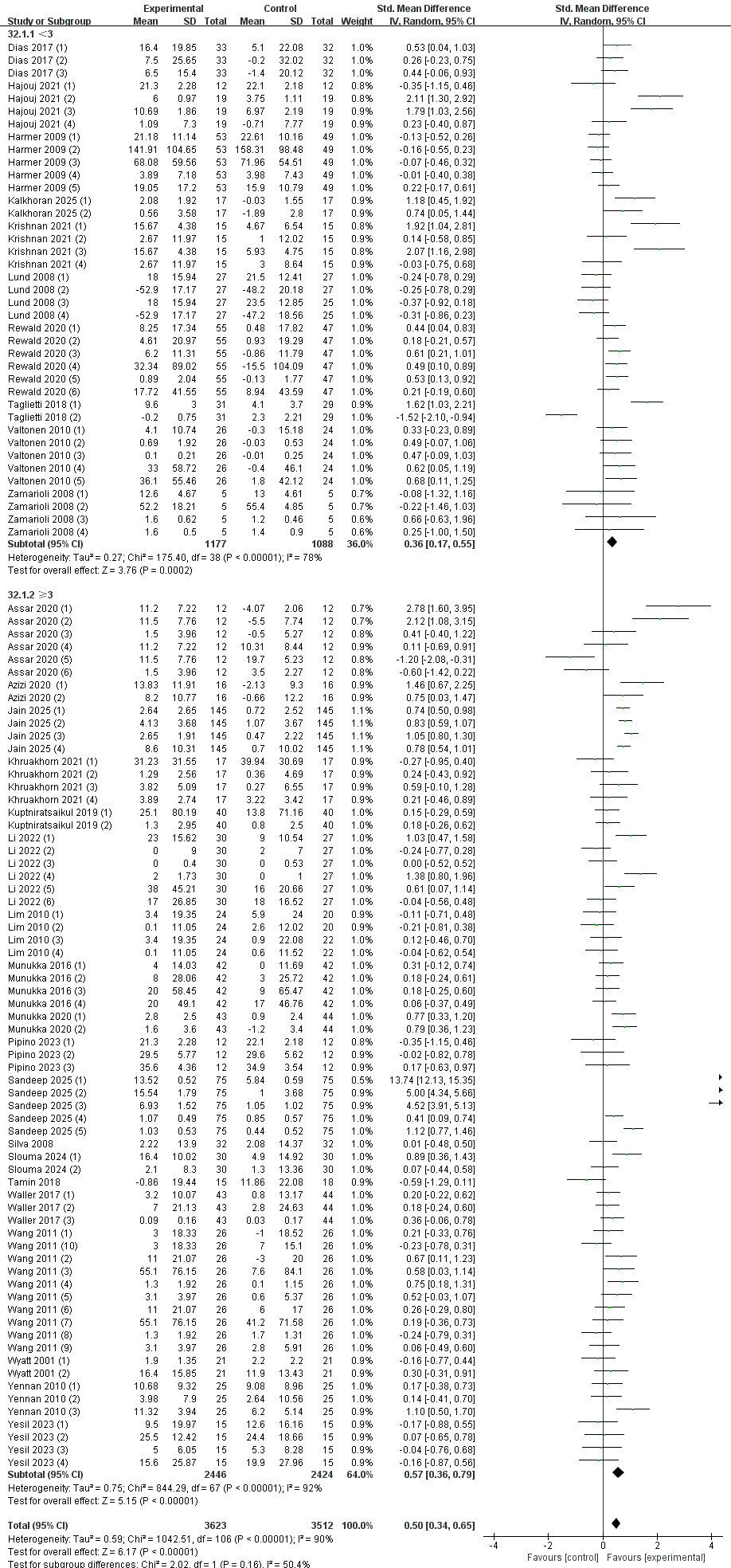


**Supplementary 4 Figure S16.** Forest plot of subgroup analyses examining the effects of aquatic rehabilitation versus control interventions on physical function, stratified by frequency


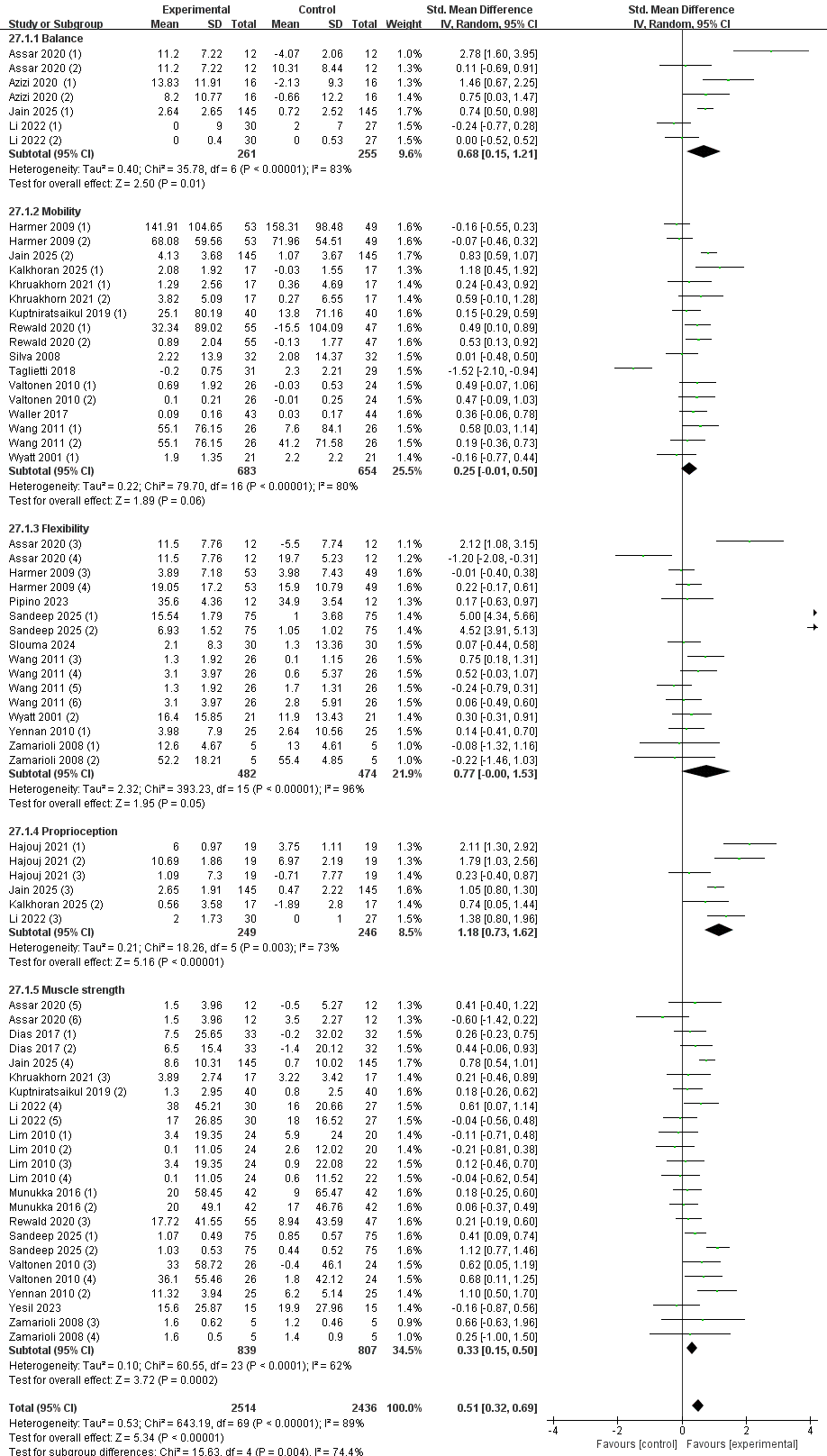


**Supplementary 4 Figure S17.** Forest plot of subgroup analyses examining the effects of aquatic rehabilitation versus control interventions on physical function, stratified by type of function


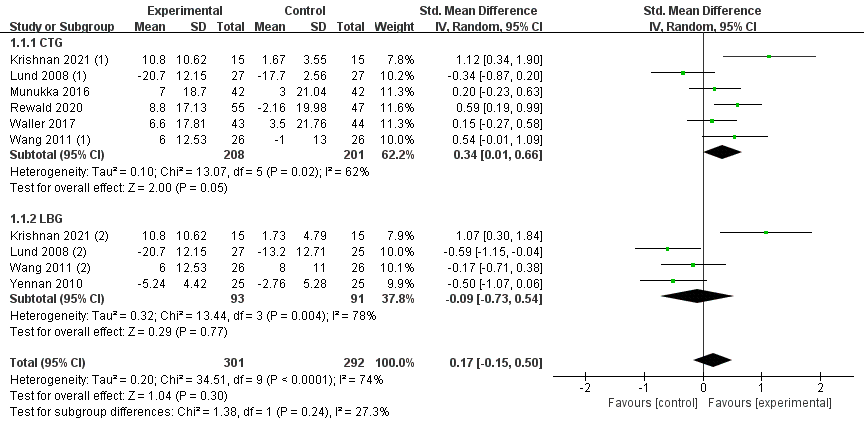


**Supplementary 4 Figure S18.** Forest plot of subgroup analyses examining the effects of aquatic rehabilitation versus control interventions on quality of life, stratified by control type

Legend: CTG conventional treatment group, LBG land-based training group.


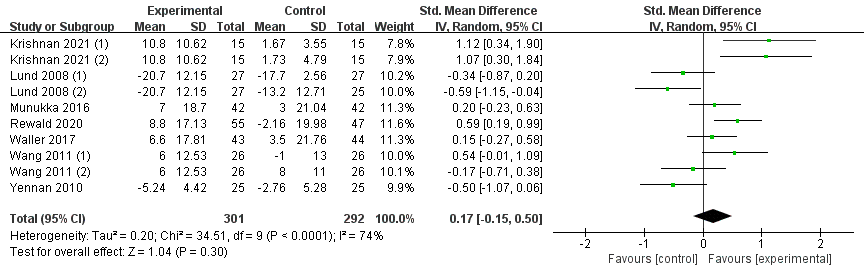


**Supplementary 4 Figure S19.** Forest plot of subgroup analyses examining the effects of aquatic rehabilitation versus control interventions on quality of life, stratified by disease type (KOA)


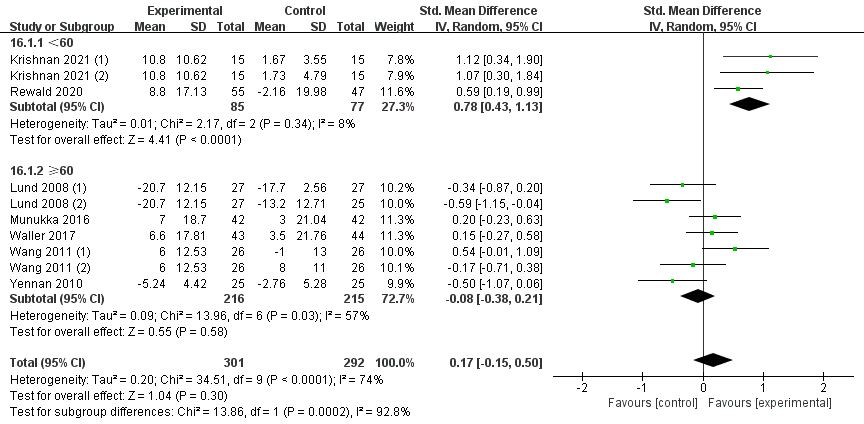


**Supplementary 4 Figure S20.** Forest plot of subgroup analyses examining the effects of aquatic rehabilitation versus control interventions on quality of life, stratified by mean age


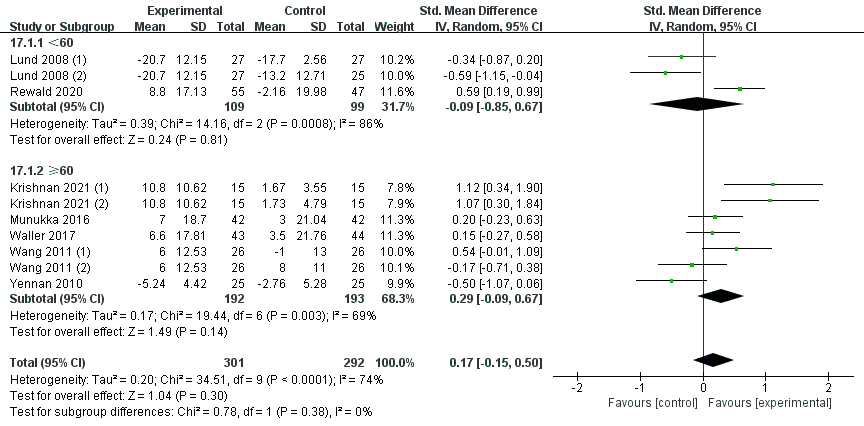


**Supplementary 4 Figure S21.** Forest plot of subgroup analyses examining the effects of aquatic rehabilitation versus control interventions on quality of life, stratified by session length


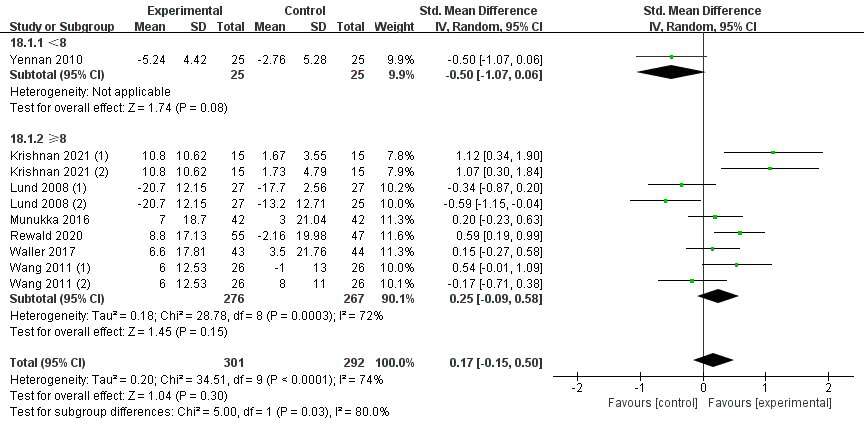


**Supplementary 4 Figure S22.** Forest plot of subgroup analyses examining the effects of aquatic rehabilitation versus control interventions on quality of life, stratified by duration


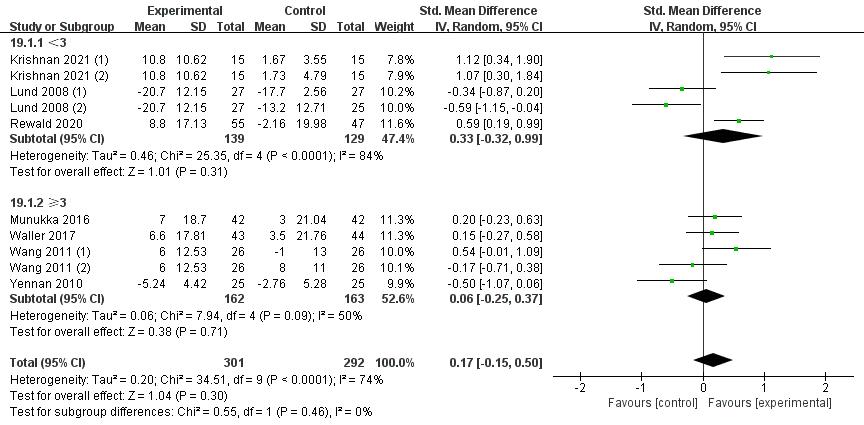


**Supplementary 4 Figure S23.** Forest plot of subgroup analyses examining the effects of aquatic rehabilitation versus control interventions on quality of life, stratified by frequency

# Risk of bias assessment and publication bias analyses


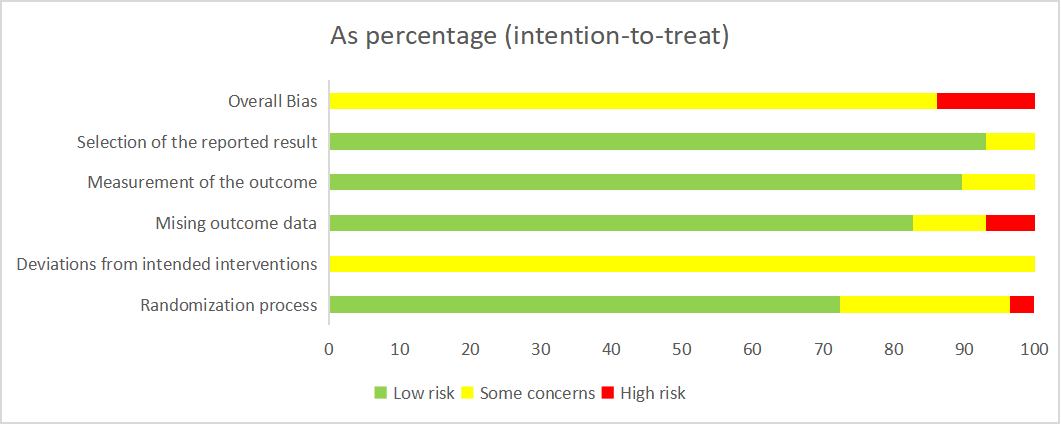


**Supplementary 5 Figure S24.** Risk of bias graph


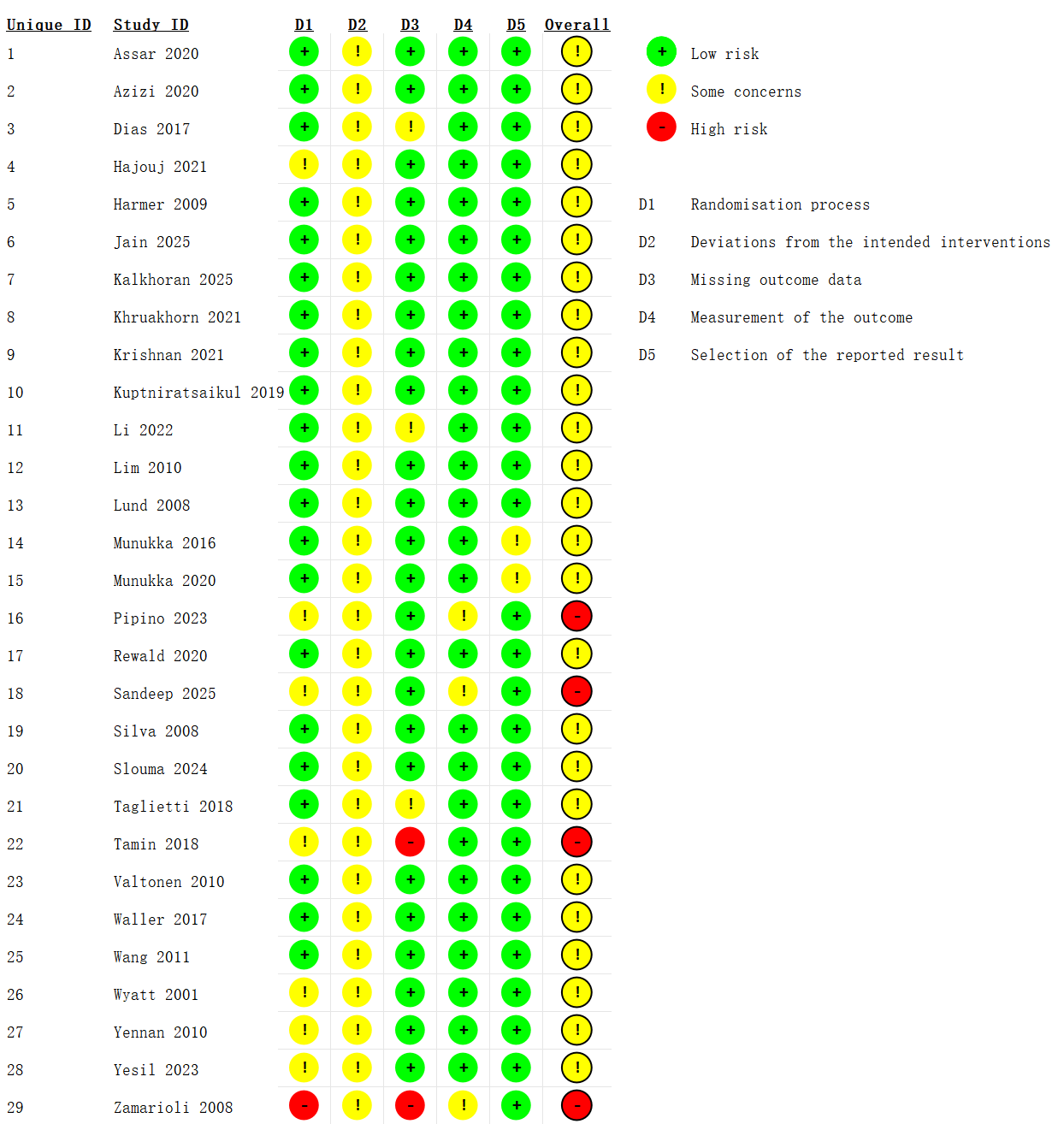


**Supplementary 5 Figure S25.** Risk of bias summary

**Supplementary 5 Table S2.** GRADE evidence profiles

| [aquatic rehabilitation ] compared to [usual care/land-based exercise] for [knee joint dysfunction]  Bibliography: | | | | | | | | | | | |
| --- | --- | --- | --- | --- | --- | --- | --- | --- | --- | --- | --- |
| Certainty assessment | | | | | | | Summary of findings | | | | |
| Participants (studies) Follow-up | Risk of bias | Inconsistency | Indirectness | Imprecision | Publication bias | Overall certainty of evidence | Study event rates (%) | | Relative effect (95% CI) | Anticipated absolute effects | |
|  |  |  |  |  |  |  | With [usual care/land-based exercise] | With [aquatic rehabilitation ] |  | Risk with [usual care/land-based exercise] | Risk difference with [aquatic rehabilitation ] |
| Symptoms | | | | | | | | | | | |
| 4463 (27 RCTs) | seriousa | very seriousb | not serious | not serious | none | ●○○○ Very lowa,b | 2203 | 2260 | - | - | SMD 0.55 SD lower (0.73 lower to 0.38 lower) |
| Physical function | | | | | | | | | | | |
| 7135 (29 RCTs) | seriousa | very seriousc | not serious | not serious | none | ●○○○ Very lowa,c | 3512 | 3623 | - | - | SMD 0.5 SD higher (0.34 higher to 0.65 higher) |
| Quality of life | | | | | | | | | | | |
| 593 (7 RCTs) | seriousa | seriousd | not serious | seriouse | none | ●○○○ Very lowa,d,e | 292 | 301 | - | - | SMD 0.17 SD higher (0.15 lower to 0.5 higher) |

CI: confidence interval; SMD: standardised mean difference

Explanations

a. All included trials involved exercise-based rehabilitation, for which blinding of participants and therapists is not feasible. Consequently, all studies were rated as “some concerns” in the domain of deviations from intended interventions under the RoB 2.0 tool. This lack of blinding increases the potential for performance bias and differential co-interventions, particularly given that several outcomes (e.g., quality of life, symptom perception) were self-reported and susceptible to participants’ expectations. Therefore, the overall risk of bias across studies was judged as moderate, leading to a one-level downgrade in the certainty of evidence.

b. Substantial heterogeneity in effect estimates (I² = 87%) indicates considerable inconsistency across studies that could not be fully explained by subgroup analyses.

c. Very high heterogeneity (I² = 90%) suggests marked inconsistency in effect sizes, likely due to variations in intervention protocols, comparator types, and patient populations.

d. Considerable heterogeneity in effect estimates (I² = 74%) persisted despite subgroup analyses, indicating inconsistency in the magnitude of effects.

e. The pooled estimate for quality of life has a 95% CI of −0.15 to 0.50, which crosses the line of no effect. Therefore, we downgraded one level for imprecision.


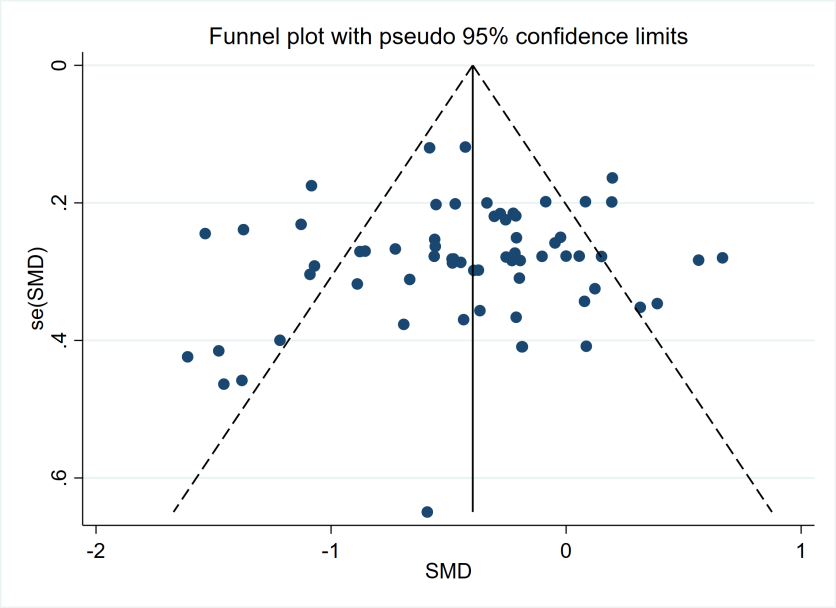


**Supplementary 5 Figure S26.** Funnel plot of composite symptoms


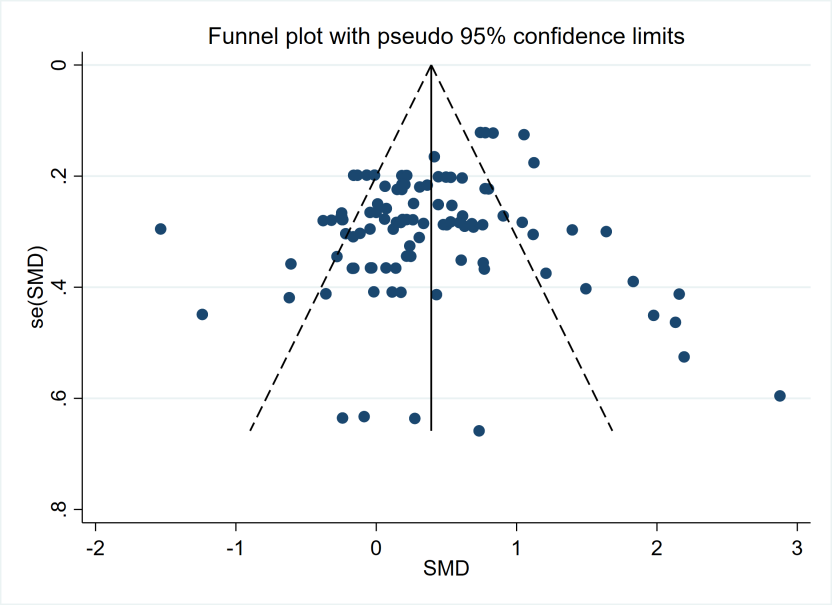


**Supplementary 5 Figure S27.** Funnel plot of composite physical function


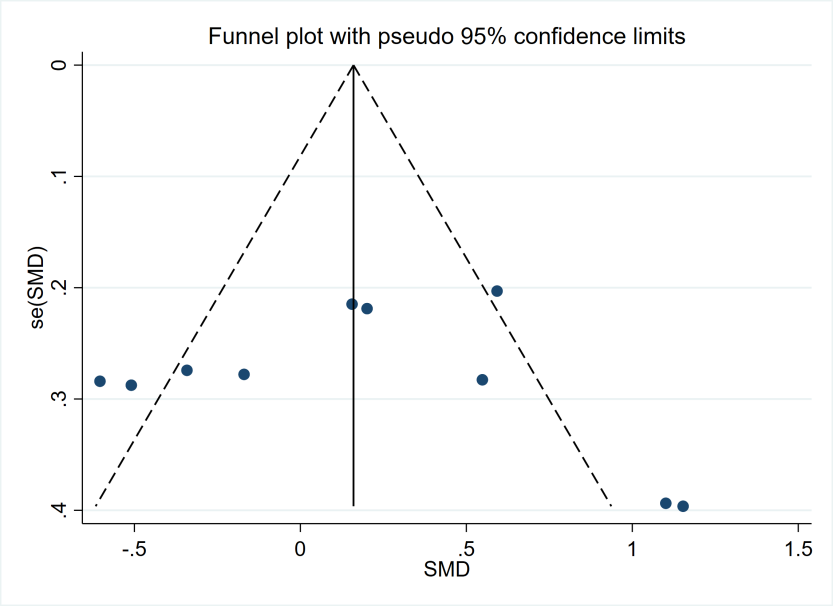


**Supplementary 5 Figure S28.** Funnel plot of quality of life
